# Bacteroidota polysaccharide utilization system for branched dextran exopolysaccharides from lactic acid bacteria

**DOI:** 10.1016/j.jbc.2023.104885

**Published:** 2023-06-02

**Authors:** Shuntaro Nakamura, Rikuya Kurata, Takashi Tonozuka, Kazumi Funane, Enoch Y. Park, Takatsugu Miyazaki

**Affiliations:** 1 Department of Bioscience, Graduate School of Science and Technology, Shizuoka University, Suruga-ku, Shizuoka, Japan; 2 Department of Agriculture, Graduate School of Integrated Science and Technology, Shizuoka University, Suruga-ku, Shizuoka, Japan; 3 Department of Applied Biological Science, Tokyo University of Agriculture and Technology, Fuchu, Tokyo, Japan; 4 Faculty of Life and Environmental Sciences, University of Yamanashi, Kofu, Yamanashi, Japan; 5 Research Institute of Green Science and Technology, Shizuoka University, Suruga-ku, Shizuoka, Japan

**Keywords:** biofilm, carbohydrate metabolism, carbohydrate-binding protein, dextran, exopolysaccharide, glucose, glycoside hydrolase, Gram-negative bacteria, polysaccharide, polysaccharide utilization locus

## Abstract

Dextran is an α-(1→6)-glucan that is synthesized by some lactic acid bacteria, and branched dextran with α-(1→2)-, α-(1→3)-, and α-(1→4)-linkages are often produced. Although many dextranases are known to act on the α-(1→6)-linkage of dextran, few studies have functionally analyzed the proteins involved in degrading branched dextran. The mechanism by which bacteria utilize branched dextran is unknown. Earlier, we identified dextranase (FjDex31A) and kojibiose hydrolase (FjGH65A) in the dextran utilization locus (FjDexUL) of a soil Bacteroidota *Flavobacterium johnsoniae* and hypothesized that FjDexUL is involved in the degradation of α-(1→2)-branched dextran. In this study, we demonstrate that FjDexUL proteins recognize and degrade α-(1→2)- and α-(1→3)-branched dextrans produced by *Leuconostoc citreum* S-32 (S-32 α-glucan). The FjDexUL genes were significantly upregulated when S-32 α-glucan was the carbon source compared with α-glucooligosaccharides and α-glucans, such as linear dextran and branched α-glucan from *L. citreum* S-64. FjDexUL glycoside hydrolases synergistically degraded S-32 α-glucan. The crystal structure of FjGH66 shows that some sugar-binding subsites can accommodate α-(1→2)- and α-(1→3)-branches. The structure of FjGH65A in complex with isomaltose supports that FjGH65A acts on α-(1→2)-glucosyl isomaltooligosaccharides. Furthermore, two cell surface sugar-binding proteins (FjDusD and FjDusE) were characterized, and FjDusD showed an affinity for isomaltooligosaccharides and FjDusE for dextran, including linear and branched dextrans. Collectively, FjDexUL proteins are suggested to be involved in the degradation of α-(1→2)- and α-(1→3)-branched dextrans. Our results will be helpful in understanding the bacterial nutrient requirements and symbiotic relationships between bacteria at the molecular level.

Bacteria produce various enzymes, including glycoside hydrolases (GHs) and polysaccharide lyases, to degrade and utilize carbohydrates depending on their habitat. Bacteria belonging to the Bacteroidota phylum possess polysaccharide utilization loci (PUL) that are involved in the systematic recognition and degradation of glycans and transport of the degradation products. PULs encode proteins such as cell surface sugar-binding proteins, sugar transporters, carbohydrate-active enzymes, such as GH and polysaccharide lyases, and transcriptional regulators (1). The study of PULs began when Salyers discovered the starch utilization system (Sus) of *Bacteroides thetaiotaomicron*, a human intestinal bacterium (2, 3, 4). Sus is composed of SusR, a transcriptional regulator, cell surface sugar-binding proteins (SusD, SusE, and SusF), TonB-dependent transporter (SusC), and GHs (SusA, SusB, and SusG) (5, 6, 7). These proteins cooperate to capture starch and break it down to oligosaccharides on the cell surface and then to D-glucose in the periplasm (8, 9, 10, 11). The sensor/regulator protein SusR recognizes maltose in the periplasm, leading to the rapid upregulation of Sus genes (6). Since then various PULs have been reported and predicted from many Bacteroidota species, including *B. thetaiotaomicron*. As of January 2023, 55,351 PULs from 1760 Bacteroidota species have been registered in the PUL database (http://www.cazy.org/PULDB/) (12). In recent years, several PULs that target fructan, chitin, hemicellulose, pectin, glycosaminoglycan, *N*-glycan, and mucin-type *O*-glycan have been reported, and many GHs with novel activities and amino acid sequences have been found (13, 14, 15, 16, 17, 18, 19, 20). Although the number of reports on PULs for plant cell wall polysaccharides and mammalian glycoconjugates increases, there are few reports of PULs targeting microbial exopolysaccharides (EPSs), except for one targeting mycobacterial D-arabinan (21).

Dextran—a polymer of α-(1→6)-linked D-glucose—is synthesized as an EPS by some lactic acid bacteria (22, 23). The structures of dextran synthesized by *Leuconostoc* and *Streptococcus* spp. vary depending on the species and strains, and branched dextran with α-(1→2)- and α-(1→3)-linkages have been reported (24, 25). Dextran from *Leuconostoc mesenteroides* NRRL B-512 is composed of 95% α-(1→6)-linkages and 5% α-(1→3)-linkages (26). In *Leuconostoc citreum* NRRL B-1299, multiple glucansucrases synthesize α-glucan with α-(1→6)-linkages in the main chain, with a relatively large proportion of α-(1→2)-branches (23%–36%) and a small number of α-(1→3)-linkages (24, 27, 28, 29, 30). *L. citreum* NRRL strain B-1355 synthesizes dextran and alternan composed of α-(1→6)- and α-(1→3)-linkages (25, 31, 32). *L. citreum* S-32 and S-64 strains, which were isolated from the lines of a sugar-manufacturing factory, synthesize branched α-glucan. ^13^C-NMR analysis revealed that S-32 α-glucan contains 30% α-(1→3)-linkages in addition to α-(1→6)-linkages; moreover, enzyme digestion analysis showed that it contains α-(1→2)-linked glucose (33, 34). By contrast, S-64 α-glucan contains 24% α-(1→2)-, 24% of α-(1→3)-, and 9% of α-(1→4)-linkages (33, 34).

Dextran-hydrolyzing enzymes have been found in some bacteria and fungi. These are divided into endo- and exo-acting enzymes. Dextranase (EC 3.2.1.11, endodextranase) randomly hydrolyzes the α-(1→6)-linkages of dextran and produces isomaltooligosaccharides with varying degrees of polymerization. According to the CAZy database (http://www.cazy.org/) (35), dextranases belong to GH families 31, 49, and 66 (GH31, GH49, and GH66) based on amino acid sequence similarity. Exo-acting dextranases are found in GH13, GH15, GH27, and GH49 families and act on the nonreducing end of dextran to generate glucose, isomaltose (IG2), or isomaltotriose (IG3) (IG*n*, isomaltooligosaccharide with degree of polymerization of *n*) (reviewed in (36)). Dextranase treatment is used in the sugar industry to prevent the loss of sucrose yield from harvested sugarcane due to the formation of dextran by microorganisms (37). An enzyme that hydrolyzes α-(1→2)-glucosyl branching of branched dextran has only been reported from *Microbacterium dextranolyticum* (formerly *Flavobacterium* sp. M-73) (38). *M. dextranolyticum* dextran α-1,2-debranching enzyme (MdDDE) was found in the cell surface and culture supernatant of this bacterium more than 40 years ago (39, 40, 41, 42). Amino acid sequence analysis of MdDDE revealed that it is a member of the GH65 family. Recombinant MdDDE could convert α-(1→2)-branched dextran produced by strains B-1299, S-32, and S-64 into glucose (34). Thus, much remains unknown about how microorganisms completely degrade branched dextran.

*Flavobacterium johnsoniae* is a Gram-negative soil bacterium that was isolated in 1947. *F*. *johnsoniae* belongs to the phylum Bacteroidota and can degrade various polysaccharides, including chitin, and proteins, such as casein and gelatin (43). *F*. *johnsoniae* has ∼200 putative carbohydrate-active enzymes (GHs, glycosyltransferases, polysaccharide lyases, and carbohydrate esterases) and ∼40 PULs (44). GH31 endodextranase (FjDex31A), identified in the *F. johnsoniae* genome [45.46], showed 35% amino acid sequence identity with other GH31 enzymes, most of which are exo-acting α-glycosidases. The endo-type activity of FjDex31A was supported by the crystallographic analysis of its active site (45). A similar phylogenetic analysis of GH65 identified kojibiose hydrolase from *F. johnsoniae* (FjGH65A, EC 3.2.1.216). FjGH65A hydrolyzes the α-(1→2)-glucosidic linkage in kojibiose (α-(1→2)-glucobiose) and longer kojioligosaccharides (46). Interestingly, *fjdex31a* and *fjgh65a* are located in the same PUL (FjDexUL), with genes encoding putative proteins involved in carbohydrate utilization; therefore, we hypothesized that FjDexUL is a PUL involved in the utilization of α-(1→2)-branched dextran. Although FjGH65A showed little activity toward α-(1→2)-branched glucan from *L. citreum* B-1299, it was active against 6-*O*-α-kojibiosylglucose and 6-*O*-α-kojitriosylglucose, which are substructures of α-(1→2)-branched dextran, comparable with kojibiose and kojitriose (46). These facts strengthened the hypothesis that FjGH65A and peripheral gene products, including FjDex31A and other GHs, work together to degrade α-(1→2)-branched dextran. In this study, we used a combination of gene expression analysis, biochemical analysis, and X-ray crystallography to evaluate the expression of FjDexUL genes and the biochemical functions of their products. Our results expand the understanding of lactic acid bacteria exopolysaccharide utilization by the Bacteroidota polysaccharide utilization system.

## Results

### Expression level of FjDexUL genes grown on branched α-glucan

**Figure 1:**
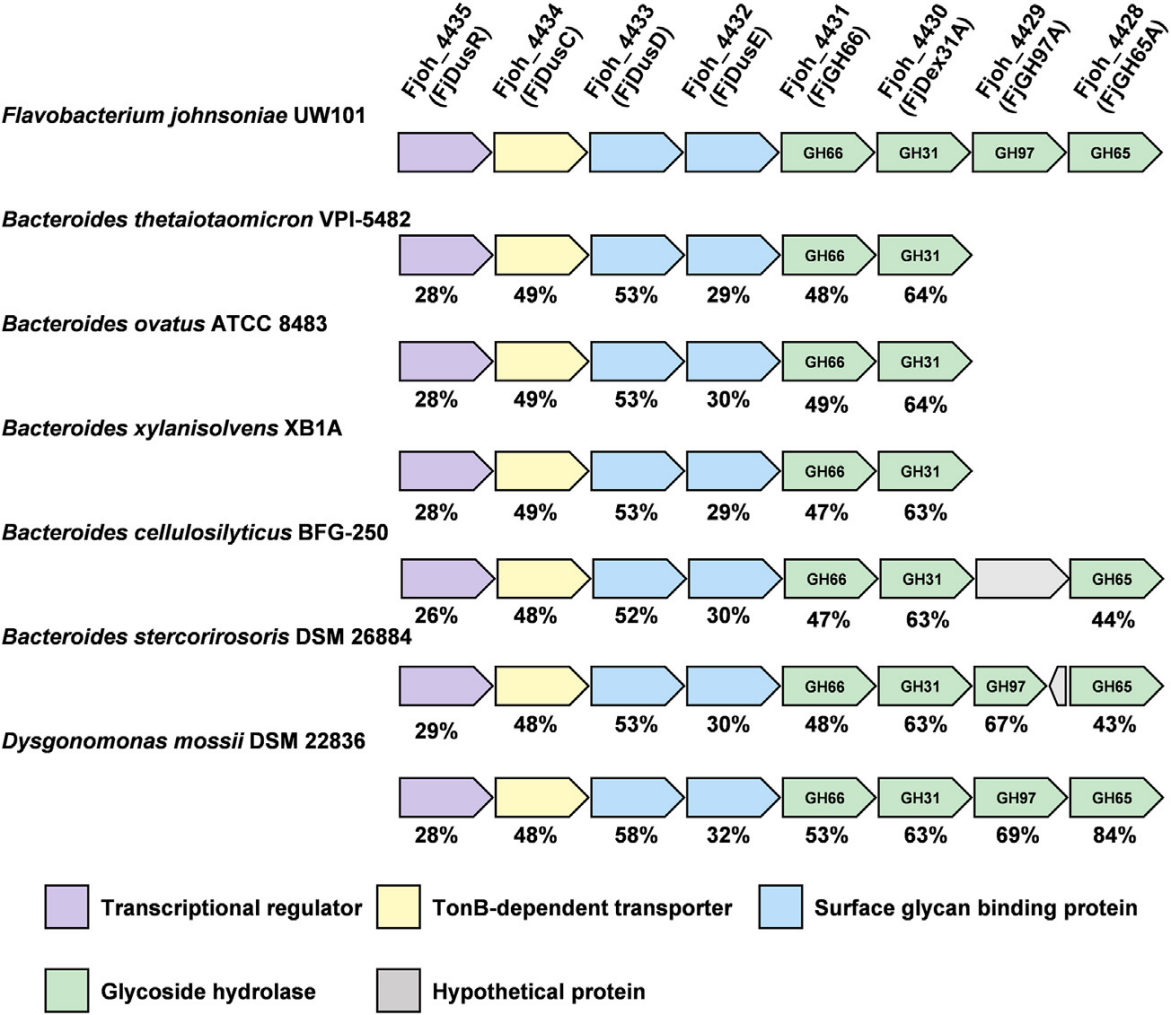
**Organization of dextran utilization loci in Bacteroidota bacteria**. FjDexUL and homologous gene loci containing genes for GH66 and GH31 proteins from Bacteroidota bacteria (*Flavobacterium johnsoniae* UW101, *Bacteroides thetaiotaomicron* VPI-5482, *Bacterides ovatus* ATCC 8483, *Bacteroides xylanisolvens* XB1A, *Bacteroides cellulosilyticus* BFG-250, *Bacteroides stercorirosoris* DSM 26884, *Dysgonomonas mossii* DSM 22836). Each PUL was referenced from the PULDB (12). Colors for genes are as follows: transcriptional regulator, *purple*; TonB-dependent transporter, *yellow*; surface sugar-binding protein, *blue*; glycoside hydrolase, *green*; hypothetical protein *gray*. The amino acid sequence homology of each protein to the FjDexUL proteins is indicated below the figure for each gene.

**Figure 2:**
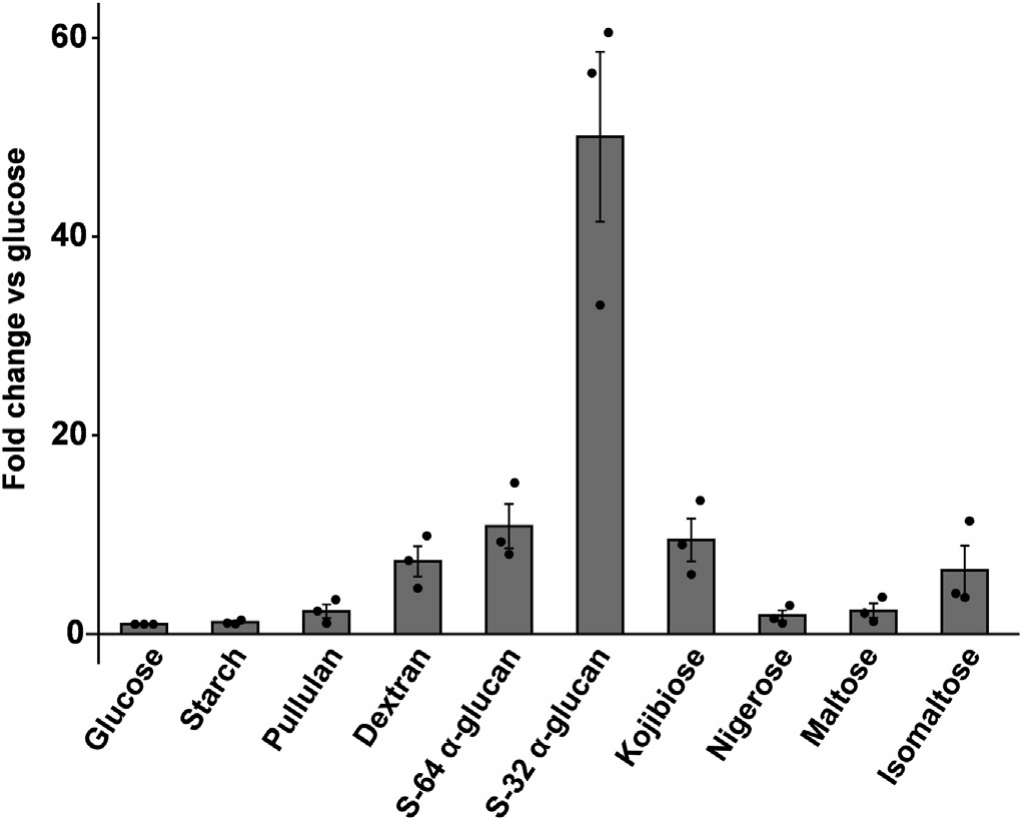
***fjdusC* expression level for different carbon sources**. The expression levels of *fjdusC* in cells grown on oligosaccharides and polysaccharides were normalized to that for glucose. The results are presented as means ± standard deviations of biological triplicates (n = 3).

FjDexUL consists of eight genes that encode proteins Fjoh_4428–Fjoh_4435: SusR homolog (Fjoh_4435, hereafter FjDusR), SusC homolog (Fjoh_4434, FjDusC), SusD homolog (Fjoh_4433, FjDusD), SusE/F homolog (Fjoh_4432, FjDusE), GH66 enzyme (Fjoh_4431, FjGH66), GH31 dextranase (Fjoh_4430, FjDex31A (44, 47)), GH97 enzyme (Fjoh_4429, FjGH97A), and GH65 kojibiose hydrolase (Fjoh_4428, FjGH65A (46)) (Fig. 1). FjGH66, FjDusD, and FjDusE were predicted to contain a type II signal peptide and are therefore, likely to be associated with the outer membrane, whereas FjDex31A, FjGH97A, and FjGH65A were predicted to have a type I signal peptide (48). The previously reported PUL contained a GH with a type I signal peptide that was primarily localized in the periplasmic space (9, 10, 13, 49). FjDex31A, FjGH97A, and FjGH65A are likely periplasmic. When PULs containing GH31 and GH66 genes were searched in PULDB (12), 70 PULs were found in Bacteroidota other than *F. johnsoniae*. However, among these PULs, GH65 genes were observed only in 11 PULs. In addition, several PULs without GH97 protein were identified (Fig. 1). To clarify which carbohydrate induces the expression of FjDexUL genes, *F. johnsoniae* was cultured in a medium with different carbon sources and the expression level of FjDexUL genes was quantified by quantitative reverse transcription PCR (qRT-PCR). The expression level of *fjdusC* dramatically increased (50 fold) when grown on S-32 α-glucan compared with glucose (Fig. 2). The cultures with S-64 α-glucan, linear dextran, kojibiose, and IG2 had an approximately 10-fold increase in *fjdusC* expression compared with glucose as the sole carbon source. By contrast, no significant change in expression levels was observed in cells grown on soluble starch, pullulan, nigerose, and maltose. Moreover, all the other genes were significantly upregulated when S-32 α-glucan was used as the carbon source (Fig. S1).

### Biochemical and structural characterization of FjGH66

**Figure 3:**
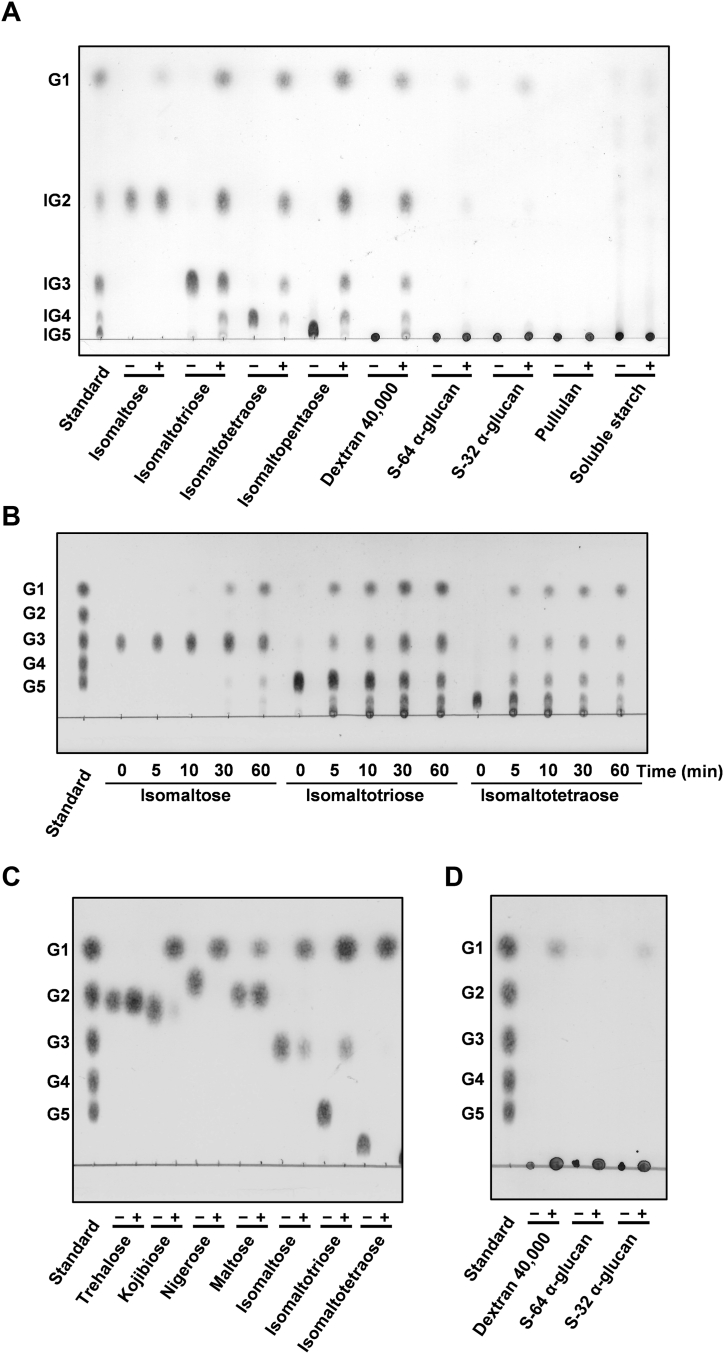
**Substrate specificity of FjDexUL GHs**. *A*, FjGH66 was incubated with 10 mM isomaltooligosaccharide and 1% α-glucans in 10 mM MES-NaOH buffer (pH 5.5). *B*, FjDex31A was incubated with 10 mM IG2, IG3, and IG4 in 10 mM MES-NaOH buffer (pH 5.5). *C* and *D*, FjGH97A was incubated with 10 mM α-glucooligosaccharides (*C*) and 1% α-glucans (*D*) in Britton–Robinson buffer (pH 7.0). All reaction mixtures were analyzed by TLC. G1, glucose; G2, maltose; G3, maltotriose; G4, maltotetraose; G5, maltopentaose.

FjGH66 is a member of the GH66 family, which contains dextranase and cycloisomaltooligosaccharide glucanotransferases (CITase) (50). FjGH66 shares 47.6% and 39.7% amino acid sequence identity with dextranases from *B. thetaiotaomicron* and *Thermoanaerobacter pseudethanolicus* (TpDex), respectively. In addition, FjGH66 was predicted to be a dextranase because there is no CITase-specific CBM35 domain insertion, which facilitates intramolecular transglycosylation to produce cycloisomaltooligosaccharides, into the catalytic module of FjGH66 (Fig. S2) (50). Recombinant FjGH66 showed the highest hydrolytic activity at pH 5.5 and 45 °C when dextran 40,000 was used as substrate (Fig. S3, *A* and *B*). FjGH66 exhibited superior hydrolytic activity toward linear dextran, and the resultant products comprise glucose, IG2, IG3, IG4, and IG5 (Table 1 and Fig. 3*A*). FjGH66 degraded IG3, IG4, and IG5 while displaying little hydrolytic activity against IG2, indicating that FjGH66 is a typical endo dextranase (Fig. 3*A*). FjGH66 showed slight hydrolytic activity toward S-32 α-glucan and S-64 α-glucan and did not hydrolyze soluble starch and pullulan (Table 1). The kinetic parameters for dextran 40,000 and dextran 200,000 have the same order of magnitude but are slightly different; therefore, its substrate-binding and hydrolytic activity may be affected by the molecular weight of the substrate (Table 2).

**Table 1:** Specific activity for the hydrolysis of various substrates by FjDexUL GHs

Enzyme	Substrate	Specific activity (μmol/min/mg)
FjGH66	Dextran 40,000 (0.5% w/v)	21.4 ± 0.47^a^
	Dextran 200,000 (0.5% w/v)	13.6 ± 0.63^a^
	S-64 α-glucan (0.5% w/v)	(9.56 ± 0.52) × 10^−2^^a^
	S-32 α-glucan (0.5% w/v)	0.153 ± 0.011^a^
	Pullulan (0.5% w/v)	N.D.^a^^b^
	Isomaltose (4 mM)	(9.12 ± 0.59) × 10^−3^^c^
	Isomaltotriose (4 mM)	(5.76 ± 0.17) × 10^−2^^c^
FjDex31A	Dextran 40,000 (0.5% w/v)	1.83 ± 0.01^a^
	Dextran 200,000 (0.5% w/v)	1.32 ± 0.02^a^
	Dextran T-2000 (0.5% w/v)^d^	1.12 ± 0.16^a^
	S-64 α-glucan (0.5% w/v)	(5.89 ± 0.91) × 10^−2^^a^
	S-32 α-glucan (0.5% w/v)	(9.30 ± 0.19) × 10^−2^^a^
	Pullulan (0.5% w/v)^d^	0.249 ± 0.008^a^
	Isomaltose (4 mM)^d^	0.104 ± 0.005^c^
	Isomaltotriose (4 mM)	4.94 ± 0.11^c^
	Isomaltotetraose (4 mM)	4.46 ± 0.36^c^
	Panose (4 mM)	0.109 ± 0.005^c^
FjGH65A	S-64 α-glucan	(3.19 ± 0.73) × 10^−2^^c^
	S-32 α-glucan	(3.55 ± 0.70) × 10^−2^^c^

a Released reducing sugar was quantified using the Somogyi–Nelson method.

b N.D., not detected.

c Released glucose was quantified using the glucose oxidase–peroxidase method.

d Values were referred from Gozu *et al*. (47).

**Table 2:** Kinetic parameters for the hydrolysis of various substrates by FjDexUL GHs

Enzyme	Substrate	*K*_m_ (mM)	*k*_cat_ (s^−1^)	*k*_cat_/*K*_m_ (s^−1^ mM^−1^)
FjGH66	Dextran 40,000	0.165 ± 0.014^a^ (6.62 ± 0.52 mg/ml)	49.1 ± 1.81	3.07 × 10^2^ (7.42 s^−1^ mg^−1^ ml)
	Dextran 200,000	(2.19 ± 0.27) × 10^−2^^a^ (4.38 ± 0.54 mg/ml)	29.7 ± 1.14	1.36 × 10^3^ (6.78 s^−1^ mg^−1^ ml)
FjDex31A	Dextran 40,000	(4.97 ± 0.70) × 10^−2^^a^ (1.99 ± 0.28 mg/ml)	3.79 ± 0.36	76.3 (1.90 s^−1^ mg^−1^ ml)
	Dextran 200,000	(1.59 ± 0.13) × 10^−2^^a^ (3.18 ± 0.26 mg/ml)	3.57 ± 0.09	2.24 × 10^2^ (0.841 s^−1^ mg^−1^ ml)
FjGH97A	Isomaltose	2.47 ± 0.34	56.9 ± 2.8	22.9
	Isomaltotriose	0.692 ± 0.072	45.5 ± 1.2	65.8
	Isomaltotetraose	0.794 ± 0.196	55.8 ± 3.7	70.3
	Kojibiose	3.46 ± 1.86	22.9 ± 5.1	6.61
	Nigerose	2.79 ± 0.71	46.5 ± 4.3	16.6
	Maltose	1.06 ± 0.21	3.00 ± 0.15	2.81

a Calculated from average molecular weight of dextran.

**Figure 4:**
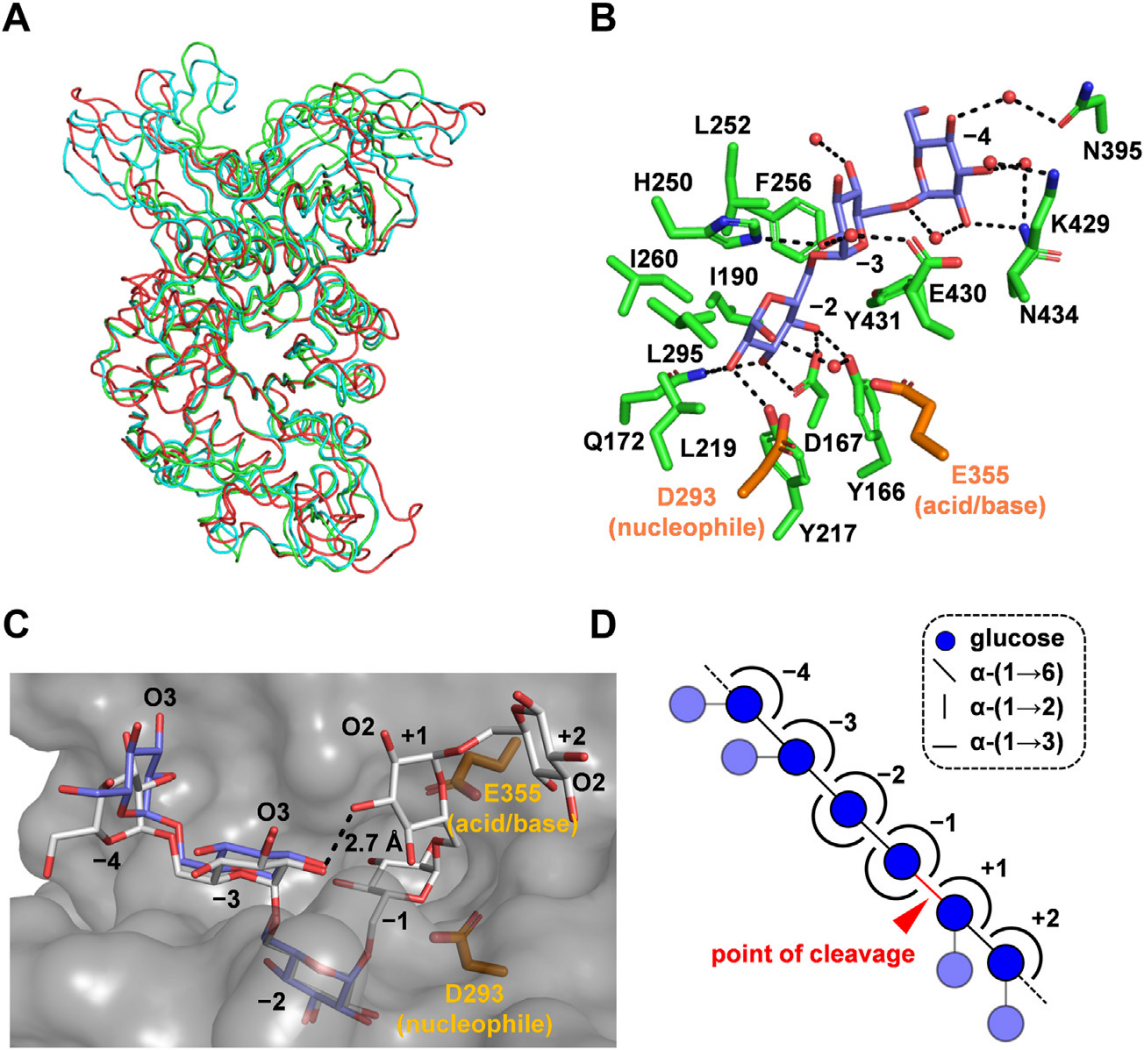
**Crystal structure of FjGH66**. *A*, superimposition of the Cα traces of FjGH66 and structure-determined GH66 enzymes. FjGH66 is shown in *green*, TpDex (PDB 5AXG) in *cyan*, and *Streptococcus mutans* dextranase (PDB 3VMN) in *red*. *B*, The IG3-bound active site of FjGH66. The side chains of amino acid residues surrounding IG3 are shown using *green stick* models. The proposed catalytic residues and IG3 are in *orange* and *slate blue*, respectively. *C*, Molecular surface of the FjGH66 catalytic cleft. FjGH66 molecule is shown in *gray*. IG3 and IG6 (derived from TpDex in complex with IG6, PDB 5AXH) are shown as stick models in *slate blue* and *white*, respectively. Solvent-exposed O2 and O3 are labeled. The intramolecular hydrogen bond in IG6 is represented by a *black dashed line*. *D*, schematic drawing of FjGH66 subsites. Sugar and linkage symbols are shown in dashed box. Acceptable α-(1→2)- and α-(1→3)-branches are shown transparently.

To elucidate the molecular mechanism of substrate recognition, the crystal structures of FjGH66 in an unliganded form and complexes with glucose, IG2, or IG3 were determined at 1.18 to 1.85 Å resolution (Table 3 and Fig. S4). All crystals, except the IG3 complex, belong to the space group *C*2, and the space group of the IG3 complex is *P*2_1_2_1_2_1_. The overall structures are almost identical (root mean squared deviation of 0.304 < Cα ≤ 0.472 Å); therefore, the following descriptions are based primarily on IG3-complex structure at 1.18 Å resolution. The FjGH66 monomer comprises three domains: N-terminal immunoglobulin fold domain (residues 41–126), (β/α)_8_-barrel fold catalytic domain (residues 127–464), and C-terminal β-sandwich domain (residues 485–586) (Fig. S4*A*). A structural homology search using the Dali server revealed that FjGH66 is most similar to TpDex (PDB 5AXG; Z score = 49.1; sequence identity = 40%) (51) and similar to GH66 enzymes, such as CITase from *Paenibacillus agaridevorans* T-3040 (PDB 3WNO; Z score = 46.1; sequence identity = 33%) (50) and dextranase from *Streptococcus mutans* (PDB 3VMP; Z score = 38.8; sequence identity = 24%) (52). The main chain of FjGH66 overlapped well with other GH66 enzymes (Fig. 4*A*). IG3 occupied the subsites −4 to −2 (subsite nomenclature is according to Davies *et al*. (53)) and the glucose residues were named Glc −4, Glc −3, and Glc −2 from the nonreducing end. All the glucose residues were in a relaxed ^4^*C*_1_ conformation. The catalytic residues (Asp293 and Glu355) and the residues forming subsites −3 to +1 were conserved between FjGH66 and GH66 enzymes (Figs. 4 and S4). To find the potential for α-(1→2)- and α-(1→3)-branched dextran acceptance of FjGH66, the FjGH66 structure was superimposed on the structure of TpDex in complex with IG6 (Fig. 4*C*). In the FjGH66 subsite −4, Phe256 and Gly253 were replaced by tryptophan and proline in TpDex, respectively, and Lys429 conformation was different from that of Lys450 of TpDex (Fig. S4, *C* and *D*). The conformations of Lys429 are identical between the ligand-free and IG3 complex structures (not shown). These may lead to different orientations of Glc −4 between the ligand complex structures of FjGH66 and TpDex (Fig. 4*C*). In the FjGH66 substrate-binding cleft, O3 of Glc −4 and Glc −3 and O2 of Glc +1 and Glc +2 were exposed to the solvent (Fig. 4, *C* and *D*). By contrast, subsites −2 and −1 are narrower than the other subsites, and O2 and O3 atoms of Glc −2 and Glc −1 are surrounded by the active site residues (Fig. 4, *C* and *D*). Therefore, α-(1→6)-linked glucose residues with α-(1→2)- and α-(1→3)-branches would not be accommodated at subsites −2 and −1. However, FjGH66 may be able to hydrolyze the α-(1→6) linkage on the nonreducing end of the α-(1→2)-glucosylated glucose residue in branched dextran due to the space available to accept an α-(1→2)-branch in the vicinity of subsite +1 (Fig. 4*D*).

**Table 3:** Data collection and refinement statistics

	FjGH66 unliganded	FjGH66/glucose	FjGH66/IG2	FjGH66/IG3	FjGH65A/IG2
Data collection					
Beamline	PF BL5A	PF BL5A	PF BL5A	PF BL5A	PF BL5A
Wavelength (Å)	1.0000	1.0000	1.0000	1.0000	1.0000
Space group	*C*2	*C*2	*C*2	*P*2_1_2_1_2_1_	*C*2
Unit cell					
*a*, *b*, *c*, (Å)	76.5, 48.4, 155.7	155.2, 48.4, 76.5	153.7, 48.0, 76.5	41.9, 91.1, 137.4	123.0, 194.2, 111.9
*β* (°)	103.775	104.405	104.640	90	116.343
Resolution range (Å)	47.26–1.85 (1.89–1.85)	47.21–1.80 (1.84–1.80)	46.86–1.80 (1.84–1.80)	45.78–1.18 (1.20–1.18)	48.54–1.56 (1.59–1.56)
Total reflections	299,826	339,941	334,449	2,165,495	2,007,211
Unique reflections	47,439	50,429	50,250	173,012	326,516
Completeness (%)	99.7 (100.0)^a^	98.3 (97.0)	99.7 (99.3)	99.9 (99.0)	98.2 (97.0)
*R*_merge_	0.112 (0.875)	0.132 (0.963)	0.121 (0.920)	0.063 (0.975)	0.072 (0.947)
CC_1/2_	0.995 (0.870)	0.997 (0.845)	0.997 (0.839)	1.000 (0.804)	0.999 (0.813)
Mean *I*/*σ*(*I*)	10.1 (1.9)	10.3 (2.1)	11.0 (2.3)	19.0 (2.5)	13.2 (2.0)
Redundancy	6.3 (6.7)	6.7 (7.0)	6.7 (6.8)	12.5 (12.0)	6.1 (6.0)
Refinement statistics					
Resolution (Å)	1.85	1.80	1.80	1.18	1.56
*R*_work_/*R*_free_	0.175/0.230	0.206/0.259	0.208/0.253	0.163/0.190	0.187/0.211
No. of atoms					
Protein	4373	4367	4398	4386	15,993
Ligand/Ion	6/6	36/1	35/1	39/3	189/−
Water	355	263	268	540	1414
Mean *B* factor (Å^2^)					
Protein	35.1	29.0	30.7	16.1	28.8
Ligand/Ion	38.1/38.1	27.0/29.9	30.9/32.3	15.4/15.7	34.5/−
Water	33.2	26.3	29.8	20.7	33.0
RMSD^b^					
Bond lengths (Å)	0.0154	0.0154	0.0083	0.0098	0.0148
Bond angles (°)	2.151	2.165	1.501	1.648	1.974
Ramachandran plot					
Favored (%)	96.49	96.32	98.01	96.69	96.60
Outliers (%)	0	0	0.18	0.18	0
Clashscore	2.91	5.80	3.34	2.42	3.27
PDB ID	8IU8	8IU9	8IUA	8IUB	8IUC

a The values for the highest resolution shells are given in parentheses.

b Root mean square deviation.

### Substrate specificity of FjDex31A on isomalto-oligosaccharides and α-glucans

FjDex31A not only hydrolyzes dextran but also catalyzes transglycosylation when IG2 is used as a substrate (45, 47). In this study, we investigated how FjDex31A acts on IG3, IG4, and branched dextran in addition to IG2 and linear dextran. FjDex31A showed hydrolytic activity against S-64 α-glucan and S-32 α-glucan as well as FjGH66 (Table 1). By contrast, FjDex31A showed glucose-producing activity against IG3 and IG4 (Table 1 and Fig. 3*B*). FjDex31A produced glucose from the initial stage of the reaction when isomaltooligosaccharides were used as substrates and catalyzed transglycosylation against IG3 and IG4, promoting the formation of longer isomaltooligosaccharides (Fig. 3*B*). The activity of FjDex31A against IG3 and IG4 was almost the same, and these specific activities were almost 47- and 3-fold higher than those against IG2 and dextran 40,000, respectively (Table 1). The *k*_cat_/*K*_m_ values for dextran 40,000 were two times higher than that for dextran 200,000 (Table 2).

### Biochemical characterization of recombinant FjGH97A

FjGH97A belongs to the GH97 family, which contains the inverting α-glucoside hydrolase or the retaining α-galactosidase (Fig. S2). FjGH97A shares 69% sequence identity with the periplasmic enzyme SusB from *B. thetaiotaomicron*. SusB is an α-glucoside hydrolase with high activity against α-(1→4)-linked oligosaccharides (54). FjGH97A showed the highest activity toward *p*-nitrophenyl α-D-glucopyranoside (pNP-Glc) at pH 7.5 and 30 °C. FjGH97A hydrolyzed α-(1→6)-, α-(1→4)-, α-(1→3)-, and α-(1→2)-linked glucobioses but not trehalose (Figs. 3, *C* and *D* and S3, *C* and *D*). Glucose was the final product of FjGH97A-treated IG3 and IG4, and only glucose production was observed from dextran 40,000 and S-32 α-glucan. These results suggest that FjGH97A is an exo-acting enzyme like other GH97 enzymes. Next, we calculated the kinetics parameters of FjGH97A substrates with α-glucoside linkages (Table 2). FjGH97A showed the highest activity against α-(1→6)-linkages, followed by nigerose, unlike SusB. FjGH97A preferred trisaccharides to disaccharides, similar to SusB. By contrast, the *k*_cat_/*K*_m_ values against kojibiose and maltose were approximately one-10th and one-20th of that against IG3, respectively. A GH97 enzyme from *Pseudoalteromonas* sp. K8 (PspAG97A) has high hydrolytic activity toward α-(1→6)-glucosidic linkages of dextran, IG2, IG3, and panose and the α-(1→2)-linkage of kojibiose, followed by maltose (55). The hydrolytic activity of PspAG97A was not significantly different between IG2 and IG3. Moreover, PspAG97A was highly active against dextran. Therefore, FjGH97A differs from PspAG97A in specificity in terms of substrate length and types of linkage other than the α-(1→6)-linkage.

### Structural insights into FjGH65A acting on the α-(1→2)-branched dextran substructure

**Figure 5:**
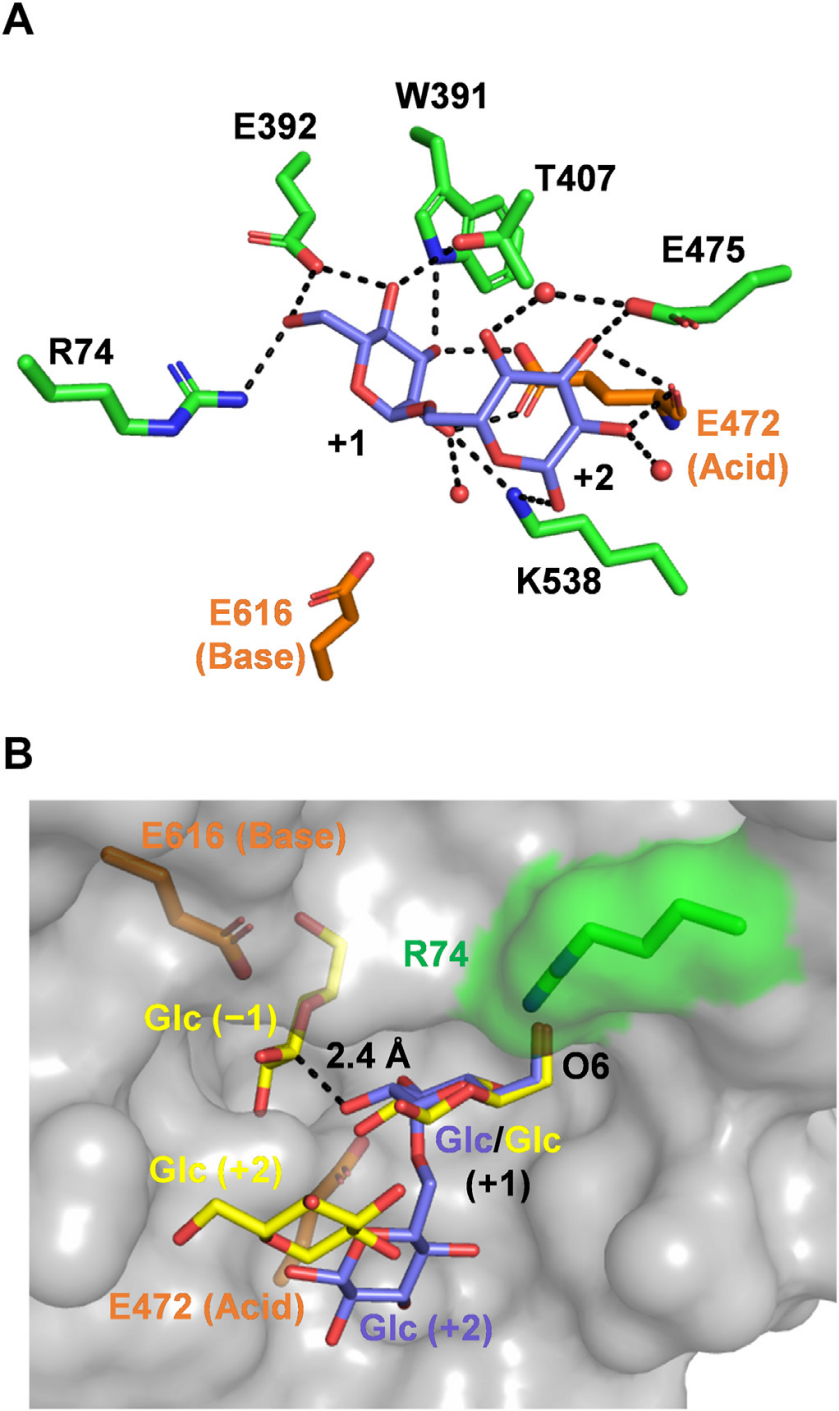
**Structure of FjGH65A complex with isomaltose**. *A*, the IG2-bound active site of FjGH65A. The side chains of amino acid residues surrounding IG2 are shown using *green stick* models. The catalytic residues and IG2 are in *orange* and *slate blue*, respectively. *B*, surface model of the FjGH65A active site. FjGH65A molecules are shown in *gray*. Three glucose molecules (*yellow stick*) in the FjGH65–glucose complex (PDB 7FE4) are superimposed. The solvent-accessible area of Arg74 is highlighted in *green*. Distance (2.4 Å) between the O2 atom of Glc +1 and the C1 atom of glucose of subsite −1 is shown as *black dashed lines*.

FjGH65A showed low activity against S-32 α-glucan and S-64 α-glucan (Table 1), although it has activity against 6-*O*-α-kojibiosylglucose, which is a substructure of α-(1→2)-branched dextran, comparable with kojibiose and kojitriose (46). To clarify how the enzyme recognizes 6-*O*-α-kojibiosylglucose, the IG2-complex structure was examined (Table 3). The 1.56-Å resolution structure enabled us to identify distinct electron densities for IG2 at subsites +1 and +2 (Figs. 5 and S5). The glucose residue at the nonreducing end of IG2 at subsite +1 (Glc +1) is recognized by Arg74, Glu392, Trp391, Thr407, and Glu472 (catalytic residue) through hydrogen bonds. The glucose residue at the reducing end at subsite +2 (Glc +2) is recognized by Glu475, Lys538, and the main chain O of catalytic Glu472 through hydrogen bonds (Fig. 5*A*). A comparison of the glucose-complex structure (PDB 7FE4 (46)), where three glucose molecules bind at subsites −1, +1, and +2, and the IG2-complex structure showed that the Glc +1 position was consistent, but Glc +2 was not. Among the hydroxy groups of Glc +1 of IG2, O2 atom of Glc +1 is the closest to the anomeric C1 atom of glucose at subsite −1 of the glucose complex at a distance of 2.4 Å (Fig. 5*B*). The reducing end of IG2 is directed toward the solvent, whereas 6-OH of Glc +1 at the nonreducing end is blocked by Arg74 (Fig. 5*B*).

### Hydrolysis of α-(1→2)- and α-(1→3)-branched dextran is orchestrated by FjDexUL GHs

**Figure 6:**
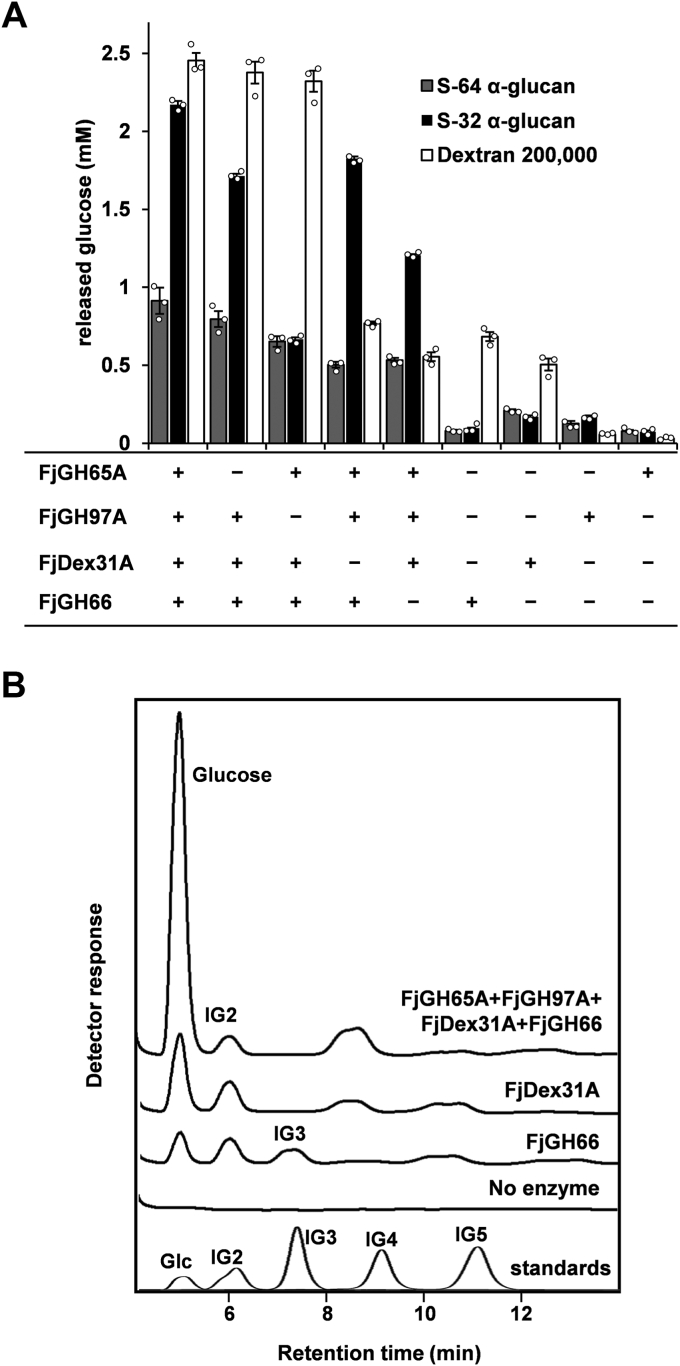
**Synergistic hydrolysis of FjDexUL GHs on branched dextran**. *A*, glucose release from dextran 200,000, S-32 α-glucan, and S-64 α-glucan after 10 min incubation with combinations of FjDexUL GHs; n = 3 independent reactions were performed with each replicate shown with the mean ± s.d. *B*, reaction products from S-32 α-glucan after 24-h incubation with FjDexUL GHs were analyzed by normal-phase HPLC using a TSK-GEL amide-80 column (4.6 mm × 250 mm; Tosoh).

Based on the substrate specificity of each FjDexUL GH examined, we investigated whether the GHs work together to degrade α-(1→2)- and α-(1→3)-branched dextran. Dextran 200,000, S-64 α-glucan, and S-32 α-glucan were treated with various combinations of FjDexUL GHs (Fig. 6*A*). Glucose production from S-64 and S-32 α-glucans by a single enzyme was low (<0.21 mM glucose produced). When S-32 α-glucan was treated with FjDex31A, FjGH97A, and FjGH65A, 1.20 mM glucose was produced. Glucose production increased 1.8-fold (2.17 mM) when FjGH66 was included in the reaction (Fig. 6*A*). Because FjGH66 generates oligosaccharides from S-32 α-glucan, which is a substrate for exo-acting enzymes (FjGH97A and FjGH65A), glucose production increased in the presence of FjGH66. Thus, FjGH66 can release α-(1→2)- and α-(1→3)-glucosylated isomaltooligosaccharides from S-32 α-glucan. The concentration of glucose released from S-32 α-glucan was 21.1% lower when FjGH65A was not included in the reaction. The most significant difference in glucose production was observed in reaction mixtures with or without FjGH97A. The addition of FjGH97A increased the amount of glucose produced from S-32 α-glucan by 1.50 mM (Fig. 6*A*). Moreover, the amount of glucose produced from S-64 α-glucan was approximately one-half that of S-32 α-glucan in the presence of all FjDexUL GHs (Fig. 6*A*). This implies that FjDexUL is more proficient in breaking down S-32 α-glucan-type structures than other α-glucans. The degradation products of S-32 α-glucan were analyzed by normal-phase high-performance liquid chromatography (HPLC). The HPLC system can separate glucose, IG2, IG3, IG4, and IG5. The retention times of glucose, IG2, IG3, IG4, and IG5 were 5, 6, 7.5, 9, and 11 min, respectively (Fig. 6*B*). When S-32 α-glucan was treated with FjGH66, peaks corresponding to glucose, IG2, and IG3 were detected; however, with FjDex31A, peaks of glucose and IG2, as well as a distinct peak at retention time 8.5 min was detected. This peak is likely a minor product of transglycosylation and did not disappear after prolonged incubation or additional enzyme treatment (data not shown). When S-32 α-glucan was treated with all four enzymes, the IG3 peak (produced by FjGH66) disappeared, and the glucose peak was significantly larger. This result suggests that exo-type enzymes play an important role in glucose production from S-32 α-glucan (Fig. 6*B*). Moreover, endodextranase, α-glucoside hydrolase, and kojibiose hydrolase synergistically degrade S-32 α-glucan into glucose.

### Binding profiles of FjDusD and FjDusE

**Figure 7:**
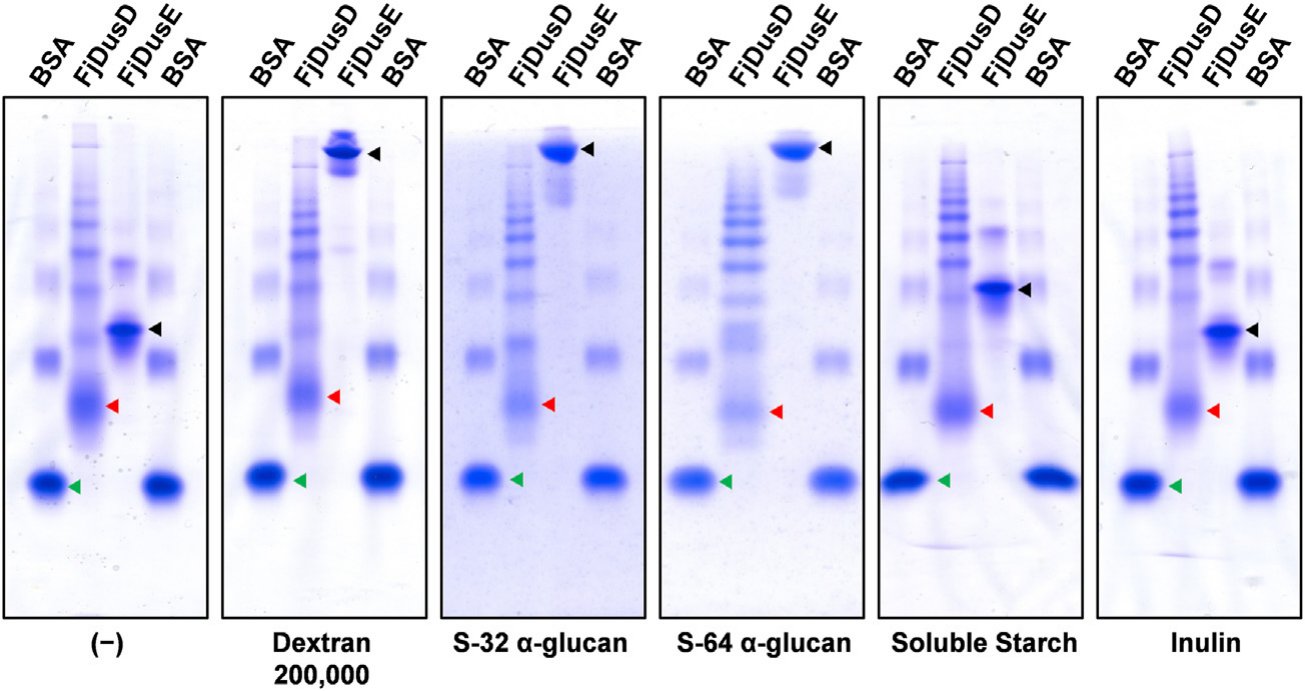
**Binding of FjDusD and FjDusE to α-glucans**. Affinity gel electrophoresis of native FjDusD and FjDusE on a gel without polysaccharide (−) and on gels with 1% (w/v) polysaccharide, dextran 200,000, S-32 α-glucan, S-64 α-glucan, soluble starch, or inulin. BSA was used as a negative control. BSA, FjDusD, and FjDusE are indicated with *green*, *red*, and *black* triangles, respectively.

FjDusD and FjDusE belong to the SusD and SusF_SusE superfamilies and share 24% and 21% sequence identity with SusD and SusF, respectively. The AlphaFold2 models of FjDusD and FjDusE are shown in Fig. S6 (56). The FjDusD model contains a tetratricopeptide repeat, similar to SusD (PDB 3CKC), and most likely has one ligand binding site (57). A comparison of the FjDusD model and crystal structures of SusD and the SusD homolog BT1762, derived from the levan utilization locus of *B. thetaiotaomicron* (PDB 6ZAZ), showed that the overall structures are similar (Fig. S7*A*). However, the conformation of the ligand-binding cleft appeared to vary based on the bound ligand (Fig. S7, *B*–*D*). The amino acid residues forming the predicted ligand-binding cleft of FjDusD were mostly conserved in closely-related homologs of FjDusD from other bacteria (Fig. S8). FjDusE has an immunoglobulin superfamily (Ig) domain and three β-sandwich carbohydrate-binding modules (FjDusEa, FjDusEb, and FjDusEc) similar to SusF (PDB 4FE9) and may have three ligand-binding sites (58). The amino acid residues responsible for ligand recognition in FjDusE were found to be partially conserved in SusF (Fig. S9). First, the affinity of these proteins toward carbohydrates was evaluated using native polyacrylamide gel electrophoresis with gels containing 0.5% (w/v) linear dextran, S-32 α-glucan, S-64 α-glucan, soluble starch, and inulin (fructan). The protein mobility profiles indicated that FjDusE binds tightly to linear dextran, S-32 α-glucan, and S-64 α-glucan and weakly to soluble starch (Fig. 7). By contrast, FjDusD displayed no significant interaction with these glucans. Next, the binding ability of FjDusD for oligosaccharides was analyzed using isothermal titration calorimetry (ITC). FjDusD showed affinity toward IG3, IG4, IG5, and IG6 but not IG2, kojibiose, kojitriose, nigerose, maltose, and maltotriose (Table 4 and Fig. S10). The *K*_d_ and Δ*G* values of FjDusD were IG5 < IG4 < IG6 < IG3. Notably, IG5 exhibited the highest binding affinity with FjDusD, based on the lowest *K*_d_ and Δ*G* values observed.

**Table 4:** Thermodynamic parameters of FjDusD binding to oligosaccharides obtained by isothermal titration calorimetry

	*K*_d_ (mM)	Δ*G* (kcal mol^−1^)	Δ*H* (kcal mol^−1^)	−*T*Δ*S* (kcal mol^−1^)
Isomaltose	N.B.	N.B.	N.B.	N.B.
Isomaltotriose	0.621 ± 0.023	−4.37 ± 0.02	−13.3 ± 0.8	8.72 ± 0.79
Isomaltotetraose	0.326 ± 0.014	−4.75 ± 0.03	−10.6 ± 0.3	6.43 ± 0.29
Isomaltopentaose	0.226 ± 0.029	−4.96 ± 0.07	−19.0 ± 3.0	8.15 ± 3.12
Isomaltohexaose	0.467 ± 0.156	−4.46 ± 0.19	−42.1 ± 3.3	37.1 ± 3.17
Kojibiose	N.B	N.B	N.B	N.B
Kojitriose	N.B.	N.B.	N.B.	N.B.
Maltose	N.B	N.B	N.B	N.B
Maltotriose	N.B.	N.B.	N.B.	N.B.
Nigerose	N.B.	N.B.	N.B.	N.B.

N.B., not binding.

## Discussion

**Figure 8:**
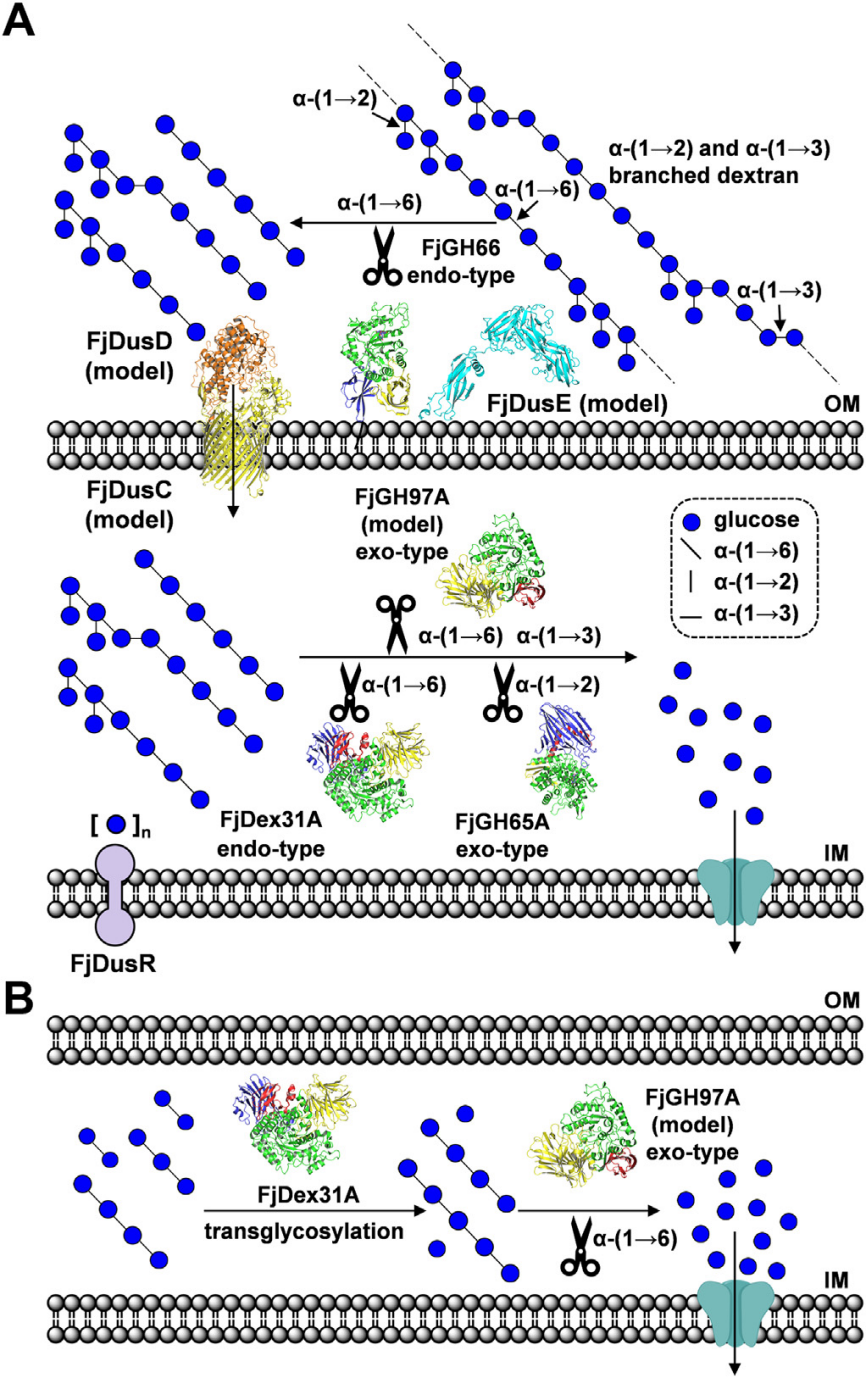
**Schematic model of branched dextran catabolism in *Flavobacterium johnsoniae*.***A*, α-(1→2)- and α-(1→3)-branched dextran is degraded into glucose sequentially by FjDexUL GHs extracellularly and in the periplasm. The uptake of oligosaccharides into the periplasm is mediated by the FjDusC–FjDusD complex, and the oligosaccharides may be recognized by FjDusR. *B*, another possible function of FjDex31A is the adjustment of the chain length of isomaltooligosaccharides, which are substrates for FjGH97A. The monomer structures of FjDex31A, FjGH65A, and FjGH66 and the AlphaFold2 models of FjDusC, FjDusD, FjDusE, and FjGH97A are shown. IM, inner membrane; OM, outer membrane.

This study demonstrated that the soil bacterium *F. johnsoniae* can identify S-32 α-glucan and α-(1→2)- and α-(1→3)-branched dextran and use FjDexUL-encoded gene products to break down S-32 α-glucan efficiently. The mechanism by which *F. johnsoniae* is proposed to utilize α-(1→2)- and α-(1→3)-branched α-glucans is shown in Figure 8. In this model, FjDusE recognizes and captures branched dextran on the bacterial surface, and FjGH66 produces α-(1→2)- and α-(1→3)-branched isomaltooligosaccharides from branched dextran. The oligosaccharide products are recognized by FjDusD and incorporated into the periplasm by the TonB-dependent transporter FjDusC. In the periplasm, FjDex31A, FjGH97A, and FjGH65A break down α-(1→2)- and α-(1→3)-branched isomaltooligosaccharides to glucose. Consistently, a similar system was reported for PULs that target other polysaccharides (1, 4, 7). The activities of a few GH66 enzymes have been analyzed against α-(1→2)- and α-(1→3)-branched dextran. Enzymatic and structural analyses of FjGH66 revealed that FjGH66 accommodates α-(1→2)- or α-(1→3)-branches at all subsites except subsites −1 and −2 (Fig. 4, *C* and *D*). GH13 α-amylases usually hydrolyze α-(1→4) linkages in the vicinity of α-(1→6)-branches in starch with difficulty; however, the catalytic clefts of SusG and *Alicyclobacillus* sp. 18,711 α-amylase were reported to accept and hydrolyze α-(1→4)-glucan with α-(1→6)-branches (59, 60). Unlike FjGH66, FjDex31A acts on not only dextran but also isomaltooligosaccharides, suggesting that FjDex31A plays a role in breaking down isomaltooligosaccharides that are degraded and incorporated through the outer cell membrane (Table 1). Notably, FjDex31A has high transglycosylation activity toward isomaltooligosaccharides. Considering that FjGH97A prefers IG3 and IG4 to IG2, the transglycosylation activity is likely used to adjust the size of isomaltooligosaccharides that can be efficiently digested by FjGH97A. A similar system of oligosaccharide chain length adjustment has been proposed in the maltooligosaccharide utilization locus of *Lactobacillus acidophilus* and other lactic acid bacteria (61). *L. acidophilus* GH13 1,4-α-glucosyltransferase (LaGH13_31B) disproportionates maltooligosaccharide length and facilitates further degradation by other GHs. FjGH97A was found to be a novel GH with high activity against isomaltooligosaccharides and nigerose, despite sharing 69% amino acid sequence identity with SusB; the details of its structure–function analysis will be published elsewhere. The IG2-complex structure of FjGH65A suggests that FjGH65A can only hydrolyze the α-(1→2)-glucosidic linkage on the nonreducing end but not on the intermediate α-(1→2)-branches in branched dextran. This is consistent with the fact that FjGH65A shows activity against the α-(1→2)-branched dextran substructures 6-*O*-α-kojibiosylglucose and 6-*O*-α-kojitriosylglucose (46).

Three distinct regulatory systems that are specific to Bacteroidota have been proposed to be responsible for the transcriptional regulation of PUL genes in Bacteroidota bacteria: SusR-like regulators, hybrid two-component systems (HTCSs), and extracytoplasmic function sigma/anti-sigma factors. These regulators perceive signals by directly interacting with oligosaccharide products through their periplasmic domains. After maltose binds to SusR, the expression of all Sus components is upregulated (6, 62). The periplasmic region of the HTCS regulator of PUL, which targets plant cell wall polysaccharides found in *B. thetaiotaomicron* and *Bacterides ovatus*, strictly recognizes the linkage type in oligosaccharides, and the favored ligands are relatively large sugar fragments, ranging from tetra-to octasaccharides (63). The HTCS regulator of the barley β-glucan PUL of *B. ovatus* was shown to exhibit high affinity for β-D-Glc-(1→4)-β-D-Glc-(1→3)-D-Glc and β-D-Glc-(1→4)-β-D-Glc-(1→3)-β-D-Glc-(1→4)-D-Glc, but not for β-D-Glc-(1→3)-β-D-Glc-(1→4)-D-Glc and laminarioligosaccharides (63). Therefore, the PUL regulator recognizes specific linkage types in oligosaccharide inducers. S-32 α-glucan remarkably upregulated the expression of FjDexUL genes. However, linear dextran, kojibiose, and IG2 did not upregulate gene expression to the same extent as S-32 α-glucan. Because the upregulation of gene expression may be influenced by factors such as ease of degradation by FjGH66 and ease of uptake by the FjDusC–FjDusD complex, it is difficult to determine the structure of the FjDusR ligand from our results. However, considering that gene expression is also induced by short oligosaccharides, IG2, and kojibiose, oligosaccharides containing α-(1→6) and α-(1→2) linkages are likely involved. Compared with S-32 α-glucan, S-64 α-glucan contains other types of linkages, such as α-(1→4) linkages and different proportions of linkages. Therefore, it is less susceptible to FjGH66, resulting in a smaller amount of oligosaccharides, which are ligands for FjDusR. Consistently, FjGH66 activity against S-64 α-glucan is slightly lower than that against S-32 α-glucan, and the synergistic effect of FjDexUL GHs on S-64 α-glucan is lower. This suggests that FjDexUL proteins strictly recognize differences in structure and branch frequency between α-(1→2)- and α-(1→3)-branched dextran.

Several studies on the functions of SusD and SusE homologs have been reported. Deletion of *susD* or mutation in the sugar-binding site of SusD inhibited the growth of *B. thetaiotaomicron* on starch (2, 57). SusD helped import degradation products from starch into the periplasm and showed affinity only for maltooligosaccharides longer than maltoheptaose (57). In this study, FjDusD had an affinity toward oligosaccharides like SusD, but no detectable affinity for dextran unlike SusD, which had an affinity for amylose (Table 3 and Fig. 7) (3). A few studies have reported SusD homologs that do not bind polysaccharides (64, 65, 66). Reverse genetic analysis and mutagenesis studies have shown that the binding of polysaccharides by SusD homologs is not essential for microbial growth in the presence of SusE homologs (7, 65, 66, 67, 68, 69). The affinity of FjDusE for linear and branched dextrans suggests that FjDusE contributes to the binding of branched dextran on the cell surface. In this study, FjDusD strictly recognized α-(1→6)-linkages. Further analysis is needed to evaluate whether FjDusD can bind isomaltooligosaccharides with α-(1→2)- or α-(1→3)-branches. The SusD homolog (BT1762) of the levan utilization locus of *B. thetaiotaomicron* binds a fructooligosaccharide with a β-(2→1)-linked branch (Fig. S7) (70). To elucidate the glucan-binding modes and physiological functions of FjDusD and FjDusE, crystallographic studies with ligands and *fjdusD* and *fjdusE* deletion strains are warranted.

Several studies on dextran with α-(1→2)- and α-(1→3)-branches have been reported; the polysaccharide is synthesized as an EPS by *Leuconostoc* spp. found in wheat sourdough and fermented products and by *Lactobacillus* sp. from the digestive tract of poultry (22, 71, 72). EPSs have been demonstrated to play a crucial role in bacterial colonization, adherence, stress resistance, host–bacterium interactions, and immunomodulation (72). This study provided insight into the mechanism of α-(1→2)- and α-(1→3)-branched dextran degradation by Gram-negative bacteria, which is different from that of α-(1→2)-branched dextran degradation by the Gram-positive bacterium *M. dextranolyticum* (34). *M. dextranolyticum* removes α-(1→2) branches extracellularly, degrades dextran, and uptakes isomaltooligosaccharides into the cytosol, whereas *F. johnsoniae* extracellularly degrades branched dextran to oligosaccharides (with branches) and hydrolyzes all linkages, including branches, in the periplasm. In addition, α-(1→2)- and α-(1→3)-branched dextran exhibited resistance to hydrolysis by digestive enzymes of both human and animal origin (73). Such resistance is attributed to the presence of branches, and the degree of resistance to hydrolysis by degrading enzymes, such as endodextranases, tends to increase with the percentage of branches. Intestinal microorganisms, such as *Bifidobacterium* and *Bacteroides* spp., can ferment α-(1→2)-branched dextran and α-glucooligosaccharides, containing α-(1→2)- and α-(1→6)-glucosidic linkages, to produce short-chain fatty acids (73, 74, 75). Given its prebiotic potential, branched dextran is a promising candidate for enhancing gut health. The findings of this study revealed that the presence or absence of GH97 and GH65 in the dextran utilization locus of *Bacteroides* is a potential indicator for intestinal bacteria that can effectively metabolize α-(1→2)- and α-(1→3)-branched dextran. Notably, several species of *Bacteroides* harbor GH97 and GH65 in their putative dextran utilization locus (Fig. 1).

In conclusion, we discovered the PUL targeting EPS from lactic acid bacteria and comprehensively investigated the gene expression and function of proteins present in FjDexUL, demonstrating that FjDexUL recognizes and degrades α-(1→2)- and α-(1→3)-branched dextran. Because bacterial species possessing both GH65 and GH97 genes in dextran utilization loci are limited, it is likely that *F. johnsoniae* acquired FjDexUL genes to utilize α-(1→2)- and α-(1→3)-branched dextran. Our results will be helpful for understanding complex polysaccharide-mediated interactions between the microbiota in nature. Furthermore, FjDexUL enzymes are expected to be a useful tool for the structural determination of branched dextran.

## Experimental procedures

### Materials and strains

All reagents used were of analytical grade and purchased from FUJIFILM Wako Pure Chemicals or Nacalai Tesque (Kyoto, Japan) unless otherwise noted. Kojibiose, kojitriose, nigerose, and IG6 were purchased from Carbosynth, and IG2, IG3, IG4, IG5, and inulin were purchased from Tokyo Chemical Industries (Tokyo, Japan). α-Glucans from *L. citreum* S-64 and *L. citreum* S-32 were prepared as described (33).

### qRT-PCR

*F. johnsoniae* cells were precultured on 0.2% yeast extract and 0.1% MgSO_4_ and inoculated in 5 ml of 0.2% yeast extract and 0.1% MgSO_4_ containing 0.5% (w/v) carbohydrates (glucose, α-glucobioses, and α-glucans). The cells were cultured for 48 h at 30 °C and harvested by centrifugation (20,640*g*, 4 °C, 5 min). Total RNA was extracted using NucleoSpin RNA plus (Takara Bio, Shiga, Japan). DNA was removed by DNase treatment using NucleoSpin RNA Clean-up (Takara Bio), and RNA quality was assessed by calculating absorbance ratios at 260/280 and 260/230 nm using the NanoDrop 2000c (Thermo Fisher Scientific). Then 1 μg RNA was used immediately for reverse transcription with PrimeScript RT Reagent Kit (Takara Bio). The expression of *fjdusR*, *fjdusC*, *fjdusD*, *fjdusE*, *fjdex31a*, *fjgh66*, *fjgh97a*, and *fjgh65a* was analyzed using quantitative PCR using THUNDERBIRD SYBR qPCR Mix (Toyobo). The gene-specific primers are listed in Table S1. The program for thermal cycling was performed using the Mx3000P system (Agilent Technologies). The cycling conditions were 15 s denaturation at 95 °C and 60 s annealing/extension at 60 °C. Data were normalized to 16S rRNA transcript levels, and change in expression level was calculated as fold-change compared with media containing glucose cultures. The experiment was performed using three independent biological replicates. Quantification of relative transcript abundance was achieved using the ΔΔCt method. The primers for quantitative PCR were designed using the Primer-BLAST server (https://www.ncbi.nlm.nih.gov/tools/primer-blast/) (76).

### Recombinant protein production and purification

Recombinant FjDex31A and FjGH65A were expressed and purified as described (46, 47). The signal peptides of FjGH66, FjGH97A, FjDusD, and FjDusE were evaluated by the SignalP server (48). The genes for FjGH66 (GenBank ID, ABQ07435.1), FjGH97A (ABQ07433.1), FjDusD (ABQ07437.1), and FjDusE (ABQ07436.1) were amplified from *F. johnsoniae* NBRC 14942 (= ATCC 17061 = UW101) by colony-direct PCR using KOD ONE DNA polymerase (Toyobo) and each pair of primers is listed in Table S1. The amplified gene product was ligated into a pET28a (+) vector (Merck Millipore) using the NheI and XhoI restriction sites. Constructed plasmids were transformed into chemically competent *E. coli* BL21 (DE3) for overexpression. All proteins were produced in 1 L Luria-Bertani medium containing 50 μg/ml kanamycin. Cells were grown at 37 °C with shaking until culture OD_600_ reached 0.6 to 0.8, at which point isopropyl β-D-thiogalactopyranoside was added at a final concentration of 0.1 mM. Protein expression was induced by overnight shaking at 20 °C. The cells were harvested, resuspended in 50 mM Tris-HCl buffer (pH 7.5) containing 300 mM NaCl and 20 mM imidazole and disrupted by sonication. The cell lysate was centrifuged to remove debris. The supernatant of the lysate was loaded onto a Ni-Sepharose excel (GE Healthcare) column, and unbound proteins were washed with the same buffer. Proteins were eluted with 50 mM Tris-HCl buffer (pH 7.5) containing 300 mM NaCl and 250 mM imidazole and then concentrated by ultrafiltration using an Amicon Ultra 30,000 molecular cutoff filter (Merck). FjGH97A was further purified by cation exchange chromatography using Mono S 5/50 GL (GE Healthcare). Equilibration was performed in 20 mM HEPES-NaOH buffer (pH 7.5) and eluted with a linear gradient of 0 to 1 M NaCl. FjGH66 was further purified with anion exchange chromatography using Mono Q 5/50 GL (GE Healthcare). Equilibration was performed in 20 mM Tris-HCl (pH 7.5) and eluted with a linear gradient of 0 to 1 M NaCl. Protein purity was determined by SDS-PAGE (Fig. S2*B*). Protein standards (molecular mass: 6500–200,000) were purchased from Nacalai Tesque. Enzyme concentrations were calculated with ExPASy ProtParam (http://web.expasy.org/protparam/) using molar absorption coefficients (FjGH65A: 18,640 M^−1^ cm^−1^, FjGH66: 115,295 M^−1^ cm^−1^, FjGH97A: 150,705 M^−1^ cm^−1^, FjDex31A: 192,935 M^−1^ cm^−1^, FjDusD: 82,405 M^−1^ cm^−1^, FjDusE: 95,715 M^−1^ cm^−1^) calculated by measuring absorbance at 280 nm with the NanoDrop 2000c (Thermo Fisher Scientific).

### Enzyme assays

To evaluate substrate specificity, FjDex31A (100 μg/ml) and FjGH66 (100 μg/ml) were incubated with 10 mM oligosaccharides and α-glucans in 10 mM MES-NaOH buffer (pH 5.5) at 30 °C. FjGH97A (100 μg/ml) was incubated with 10 mM oligosaccharides and α-glucans in Britton–Robinson buffer (pH 7.0) at 30 °C. Reaction mixtures were analyzed by thin-layer chromatography (TLC): 1.5 μl of each sample was spotted on TLC plates of Silica Gel 60 (Merck) and developed with nitromethane:1-propanol:water = 4:10:3. The plates were dried, sprayed with 5% (v/v) sulfuric acid–methanol solution, and charred at 110 °C.

To evaluate the specific activities of FjDex31A and FjGH66 against IG2, IG3, IG4, panose, dextran 40,000, dextran 200,000, pullulan, S-64 α-glucan, and S-32 α-glucan were used. The released reducing sugar was quantified using the Somogyi–Nelson method (77, 78) and released glucose was quantified with the glucose oxidase–peroxidase method using Glucose C-II Test Kit (Wako Pure Chemicals). The enzyme reaction volume was a 50-μL reaction mixture containing 100 μg/ml FjDex31A or 100 μg/ml FjGH66, 4 mM oligosaccharide or 0.5% (w/v) polysaccharide, and 50 mM MES-NaOH buffer (pH 5.5) at 30 °C. The reaction was performed for 2 to 30 min and stopped by boiling for 5 min. To determine optimal pH, the reaction was performed in Britton–Robinson buffer (pH 2.0–9.0) containing 10 μg/ml FjGH97A for 10 mM pNP-Glc or 10 μg/ml FjGH66 for 0.5% (w/v) dextran 40,000 at 30 °C for 10 min. In the case of FjGH97A, the optimal temperature was 20 °C–70 °C for 10 μg/ml FjGH97A and 10 mM pNP-Glc in Britton–Robinson buffer (pH 7.0) for 10 min. In the case of FjGH66, the optimal temperature was 25 °C–70 °C for 10 μg/ml FjGH66 and 0.5% (w/v) dextran 40,000 in 50 mM MES-NaOH buffer (pH 5.5) for 10 min. To determine the kinetic parameters, initial velocities of at least five concentrations for each substrate were measured and fitted to the Michaelis–Menten equation using Enzyme_Kinetics_Calculator (79). The enzyme concentrations used were 10 μg/ml (0.12 μM) FjGH97A, 100 μg/ml (1.5 μM) FjDex31A, and 10 μg/ml (0.15 μM) FjGH66 against 0.1 to 20 mM IG2, IG3, kojibiose, nigerose, and maltose and 0.1%–2% dextran 40,000 in 50 mM MES-NaOH buffer (pH 5.5). Because nigerose is degraded by heat treatment under neutral conditions (80), the reaction was stopped by adding 0.5 M Na_2_CO_3_ when nigerose was used as a substrate.

### Synergistic activities of FjDexUL GHs on branched dextran

To determine if FjDexUL GHs function synergistically, a combination of 100 μg/ml FjDexUL GHs was used to hydrolyze dextran 200,000, S-64 α-glucan, and S-32 α-glucan at 0.5% (w/v) final concentration, pH 5.5, and 30 °C for 10 min or 24 h. The released glucose was determined by the glucose oxidase–peroxidase method and degradation products were detected using HPLC. The reaction mixtures were then applied to a TSK-GEL amide-80 column (4.6 mm × 250 mm; Tosoh) immediately after incubation and eluted with 60% (v/v) acetonitrile at a flow rate of 1.0 ml/min at 30 °C. The reaction products were detected using a refractive index detector (RID-10A, Shimadzu, Kyoto, Japan).

### Crystallization and structure determination

FjGH65A (30 mg/ml in 20 mM sodium citrate buffer, pH 6.0, and 150 mM NaCl) was crystallized as described (46) and then soaked with reservoir solutions containing 10 mM IG2 for 1 min. FjGH66 (20 mg/ml in 10 mM HEPES-NaOH buffer; pH 7.0) was crystallized at 20 °C using the hanging drop vapor diffusion method, where 1 μl of protein was mixed with the same volume of reservoir solution, consisting of 0.1 M Tris-HCl buffer (pH 8.5 and 9.0), 0.2 M lithium sulfate, and 20% (v/v) PEG 4000. For the glucose- and IG2-complexes, crystals of FjGH66 were soaked with reservoir solutions containing 10 mM glucose or IG2 for 1 min. For the IG3-complex, FjGH66 was co-crystallized using a reservoir solution containing 10 mM IG3. All crystals were cryoprotected with 20% (v/v) glycerol in reservoir solution and flash-frozen in liquid nitrogen. Diffraction data were collected at the BL5A beamline (Photon Factory). The data were processed with XDS and then scaled using AIMLESS, as implemented in the CCP4i2 package (81, 82). The initial phase was determined with the molecular replacement method using MOLREP (83) with PDB 7FE3 and the AlphaFold2 model as a search model for the FjGH65A–IG2 complex and FjGH66, respectively (56). Manual model building was performed using COOT (84), and refinement was performed using REFMAC5 (85). Molecular images were made using PyMOL (Schrödinger LLC). Structural similarity searches were performed using the Dali server (86). Table 4 summarizes data collection and refinement statistics. All AlphaFold2 models were downloaded from the AlphaFold Protein Structure Database (56, 87).

### Affinity gel electrophoresis

Running gels were composed of 8% polyacrylamide gel (acrylamide:*N,N′*-methylenebisacrylamode = 30:1) and 0.5% (w/v) polysaccharide. FjDusD (7.5 μg), FjDusE (5 μg), and bovine serum albumin (5 μg) were loaded onto gels and electrophoresed under nondenaturing conditions using 0.1 M Tris-HCl (pH 7.8) as the anode buffer and 0.068 M glycine-Tris-HCl (pH 8.9) as the cathode buffer at 80 V for 3 h at room temperature. Proteins were visualized by staining with Coomassie Brilliant Blue.

### Isothermal titration calorimetry

All ITC experiments were performed using MicroCal iTC200 (Malvern Panalytical Ltd) calibrated to 25 °C. All titrations were performed in 10 mM HEPES-NaOH buffer (pH 7.0). Purified FjDusD and FjDusE (0.1 mM each) were loaded into the sample cell, and the syringe was loaded with 5 mM IG5 and IG6 and 10 mM kojibisoe, kojitriose, nigerose, maltose, maltotriose, IG2, IG3, and IG4. An initial injection of 0.2 μl was followed by 19 injections of 2 μl spaced 150 s apart, with an injection duration of 4 s. Titration results were analyzed using MicroCal Origin ITC (Malvern Panalytical Ltd,). Data were fitted to a standard one-site binding model (n = 1), with ligand concentration as a variable. The average and standard error were calculated using data from at least three tests.

## Data availability

The atomic coordinates and structure factors were deposited in the Worldwide Protein Data Bank (http://wwpdb.org/) under the accession codes 8IU8, 8IU9, 8IUA, 8IUB, and 8IUC. All other data are contained within the article.

## Supporting information

This article contains supporting information.

## Conflicts of interest

The authors declare that they have no conflicts of interest with the contents of this article.

## References

[1] Grondin J.M., Tamura K., Déjean G., Abbott D.W., Brumer H. (2017). Polysaccharide utilization loci: Fueling microbial communities. J. Bacteriol..

[2] Anderson K.L., Salyers A.A. (1989). Biochemical evidence that starch breakdown by *Bacteroides thetaiotaomicron* involves outer membrane starch-binding sites and periplasmic starch-degrading enzymes. J. Bacteriol..

[3] Shipman J.A., Berleman J.E., Salyers A.A. (2000). Characterization of four outer membrane proteins involved in binding starch to the cell surface of *Bacteroides thetaiotaomicron*. J. Bacteriol..

[4] Martens E.C., Koropatkin N.M., Smith T.J., Gordon J.I. (2009). Complex glycan catabolism by the human gut microbiota: the bacteroidetes sus-like paradigm. J. Biol. Chem..

[5] Tancula E., Feldhaus M.J., Bedzyk L.A., Salyers A.A. (1992). Location and characterization of genes involved in binding of starch to the surface of *Bacteroides thetaiotaomicron*. J. Bacteriol..

[6] D’Elia J.N., Salyers A.A. (1996). Effect of regulatory protein levels on utilization of starch by Bacteroides thetaiotaomicron. J. Bacteriol..

[7] Foley M.H., Cockburm D.W., Koropatkin N.M. (2016). The Sus operon: a model system for starch uptake by the human gut bacteroidetes. Cell Mol. Life Sci..

[8] Shipman J.A., Cho K.H., Siegel H.A., Salyers A.A. (1999). Physiological characterization of SusG, an outer membrane protein essential for starch utilization by Bacteroides thetaiotaomicron. J. Bacteriiol..

[9] Smith K.A., Salyers A.A. (1991). Characterization of a neopullulanase and an α-glucosidase from *Bacteroides thetaiotaomicron* 95-1. J. Bacteriol..

[10] D'Elia J.N., Salyers A.A. (1996). Contribution of a neopullulanase, a pullulanase, and an α-glucosidase to growth of *Bacteroides thetaiotaomicron* on starch. J. Bacteriol..

[11] Reeves A.R., D'Elia J.N., Frias J., Salyers A.A. (1996). A *Bacteroides thetaiotaomicron* outer membrane protein that is essential for utilization of maltooligosaccharides and starch. J. Bacteriol..

[12] Terrapon N., Lombard V., Drula É., Lapébie P., Al-Masaudi S., Gilbert H.J. (2018). Puldb: The expanded database of polysaccharide utilization loci. Nucl. Acids Res..

[13] Sonnenburg E.D., Zheng H., Joglekar P., Higginbottom S.K., Firbank S.J., Bolam D.N. (2010). Specificity of polysaccharide use in intestinal bacteroides species determines diet-induced microbiota alterations. Cell.

[14] Larsbrink J., Zhu Y., Kharade S.S., Kwiatkowski K.J., Eijsink V.G.H., Koropatkin N.M. (2016). A polysaccharide utilization locus from *Flavobacterium johnsoniae* enables conversion of recalcitrant chitin. Biotechnol. Biofuels.

[15] Rogowski A., Briggs J.A., Mortimer J.C., Tryfona T., Terrapon N., Lowe E.C. (2015). Glycan complexity dictates microbial resource allocation in the large intestine. Nat. Commun..

[16] Naas A.E., Mackenzie A.K., Mravec J., Schückel J., Willats W.G.T., Eijsink V.G.H. (2014). Do rumen Bacteroidetes utilize an alternative mechanism for cellulose degradation?. mBio.

[17] Ndeh D., Rogowski A., Cartmell A., Luis A.S., Baslé A., Gray J. (2017). Complex pectin metabolism by gut bacteria reveals novel catalytic functions. Nature.

[18] Cartmell A., Lowe E.C., Baslé A., Firbank S.J., Ndeh D.A., Murray H. (2017). How members of the human gut microbiota overcome the sulfation problem posed by glycosaminoglycans. Proc. Natl. Acad. Sci. U. S. A..

[19] Crouch L.I., Urbanowicz P.A., Baslé A., Cai Z.P., Liu L., Voglmeir J. (2022). Plant *N*-glycan breakdown by human gut *Bacteroides*. Proc. Natl. Acad. Sci. U. S. A..

[20] Martens E.C., Chiang H.C., Gordon J.I. (2008). Mucosal glycan foraging enhances fitness and transmission of a saccharolytic human gut bacterial symbiont. Cell Host Microbe.

[21] Al-Jourani O., Benedict S.T., Ross J., Layton A.J., van der Peet P., Marando V.M. (2023). Identification of D-arabinan-degrading enzymes in mycobacteria. Nat. Commun..

[22] Jeanes A., Haynes W.C., Wilham C.A., Rankin J.C., Melvin E.H., Austin M.J. (1954). Characterization and classification of dextrans from ninety-six strains of bacteria. J. Am. Chem. Soc..

[23] Li X., Wang X., Meng X., Diikhuizen L., Liu W. (2020). Structures, physico-chemical properties, production and (potential) applications of sucrose-derived α-d-glucans synthesized by glucansucrases. Carbohydr. Polym..

[24] Kim D., Robyt J.F. (1995). Dextransucrase constitutive mutants of *Leuconostoc mesenteroides* B-1299. Enzyme Microb. Technol..

[25] Smith M.R., Zahnley J., Goodman N. (1994). Glucosyltransferase mutants of *Leuconostoc mesenteroides* NRRL B-1355. Appl. Environ. Microbiol..

[26] Sloan W.J., Alexander B.H., Lohmar R.L., Wolff I.A., Rist C.E. (1954). Determination of dextran structure by periodate oxidation techniques. J. Am. Chem. Soc..

[27] Gangoiti J., Pijning T., Dijkhuizen L. (2018). Biotechnological potential of novel glycoside hydrolase family 70 enzymes synthesizing α-glucans from starch and sucrose. Biotechnol. Adv..

[28] Seymour F.R., Chen E.C.M., Bishop S.H. (1979). Methylation structural analysis of unusual dextrans by combined gas-liquid chromatography-mass spectrometry. Carbohydr. Res..

[29] Kobayashi M., Shishido K., Kikuchi T., Matsuda K. (1973). Fractionation of the *Leuconostoc mesenteroides* NRRL B-1299 dextran and preliminary characterization of the fractions. Agric. Biol. Chem..

[30] Kobayashi M., Shishido K., Kikuchi T., Matsuda K. (1973). Methylation analysis of fractions from the *Leuconostoc mesenteroides* NRRL B-1299 dextran. Agric. Biol. Chem..

[31] Misaki A., Torii M., Sawai T., Goldstein I.J. (1980). Structure of the dextran of *Leuconostoc mesenteroides* B-1355. Carbohydr. Res..

[32] Argüello-Morales M.A., Remaud-Simeon M., Pizzut S., Sarçabal P., Willemot R., Monsan P. (2000). Sequence analysis of the gene encoding alternansucrase, a sucrose glucosyltransferase from *Leuconostoc mesenteroides* NRRL B-1355. FEMS Microbiol. Lett..

[33] Funane K., Matsuo T., Ono H., Ishii T., Gibu S., Tokashiki T. (2003). Characterization of glucans and glucansucrases from novel *Leuconostoc* strains (including sp. S-51). J. Appl. Glycosci..

[34] Miyazaki T., Tanaka H., Nakamura S., Dohra H., Funane K. (2023). Identification and characterization of dextran α-1,2-debranching enzyme from *Microbacterium dextranolyticum*. J. Appl. Glycosci..

[35] Drula E., Garron M.L., Dogan S., Lombard V., Henrissat B., Terrapon N. (2022). The carbohydrate-active enzyme database: functions and literature. Nucl. Acids Res..

[36] Miyazaki T. (2023). Glycoside hydrolases active on microbial exopolysaccharide α-glucans: structures and function. Essays Biochem..

[37] Rodríguez Jiménez E. (2009). Dextranase in sugar industry: a review. Sugar Tech..

[38] Yokota A., Takeuchi M., Weiss N. (1993). Proposal of two new species in the genus *Microbacterium: microbacterium dextranolyticum* sp. nov. And *Microbacterium aurum* sp. nov. Int. J. Syst. Bacteriol..

[39] Kobayashi M., Mitsuishi Y., Matsuda K. (1978). Pronounced hydrolysis of highly branched dextrans with a new type of dextranase. Biochem. Biophys. Res. Commun..

[40] Mitsuishi Y., Kobayashi M., Matsuda K. (1979). Dextran α-1,2 debranching enzyme from *Flavobacterium* sp. M-73: its production and purification. Agric. Biol. Chem..

[41] Mitsuishi Y., Kobayashi M., Matsuda K. (1980). Dextran α-(1→2)-debranching enzyme from *Flavobacterium* Sp. M-73. Properties and mode of action. Carbohydr. Res..

[42] Mitsuishi Y., Kobayashi M., Matsuda K. (1984). Structures of three α-D-(1→2)-branched oligosaccharides isolated from *Leuconostoc mesenteroides* NRRL B-1299 dextran. Carbohydr. Res..

[43] Stanier R.Y. (1947). Studies on nonfruiting myxobacteria: i. *Cytophaga johnsonae*, n.sp., a chitin-decomposing myxobacterium. J. Bacteriol..

[44] McBride M.J., Xie G., Martens E.C., Lapidus A., Henrissat B., Rhodes R.G. (2009). Novel features of the polysaccharide-digesting gliding bacterium *Flavobacterium johnsoniae* as revealed by genome sequence analysis. Appl. Environ. Microbiol..

[45] Tsutsumi K., Gozu Y., Nishikawa A., Tonozuka T. (2020). Structural insights into polysaccharide recognition by *Flavobacterium johnsoniae* dextranase, a member of glycoside hydrolase family 31. FEBS J..

[46] Nakamura S., Nihira T., Kurata R., Nakai H., Funane K., Park E.Y. (2021). Structure of a bacterial α-1,2-glucosidase defines mechanisms of hydrolysis and substrate specificity in GH65 family hydrolases. J. Biol. Chem..

[47] Gozu Y., Ishizaki Y., Hosoyama Y., Miyazaki T., Nishikawa A., Tonozuka T. (2016). A glycoside hydrolase family 31 dextranase with high transglucosylation activity from *Flavobacterium johnsoniae*. Biosci. Biotechnol. Biochem..

[48] Armenteros J.J.A., Tsirigos K.D., Sønderby C.K., Petersen T.N., Winther O., Brunak S. (2019). SignalP 5.0 improves signal peptide predictions using deep neural networks. Nat. Biotechnol..

[49] Joglekar P., Sonnenburg E.D., Higginbottom S.K., Earle K.A., Morland C., Shapiro-Ward S. (2018). Genetic variation of the SusC/SusD homologs from a polysaccharide utilization locus underlies divergent fructan specificities and functional adaptation in *Bacteroides thetaiotaomicron* strains. mSphere.

[50] Suzuki N., Fujimoto Z., Kim Y.M., Momma M., Kishine N., Suzuki R. (2014). Structural elucidation of the cyclization mechanism of α-1,6-glucan by *Bacillus circulans* T-3040 cycloisomaltooligosaccharide glucanotransferase. J. Biol. Chem..

[51] Suzuki N., Kishine N., Fujimoto Z., Sakurai M., Momma M., Ko J.A. (2016). Crystal structure of thermophilic dextranase from *Thermoanaerobacter pseudethanolicus*. J. Biochem..

[52] Suzuki N., Kim Y.M., Fujimoto Z., Momma M., Okuyama M., Mori H. (2012). Structural elucidation of dextran degradation mechanism by *Streptococcus mutans* dextranase belonging to glycoside hydrolase family 66. J. Biol. Chem..

[53] Davies G.J., Wilson K.S., Henrissat B. (1997). Nomenclature for sugar-binding subsites in glycosyl hydrolases. Biochem. J..

[54] Kitamura M., Okuyama M., Tanzawa F., Mori H., Kitago Y., Watanabe N. (2008). Structural and functional analysis of a glycoside hydrolase family 97 enzyme from *Bacteroides thetaiotaomicron*. J. Biol. Chem..

[55] Li W., Fan H., He C., Zhang X., Wang X., Yuan J. (2016). PspAG97A: a halophilic α-glucoside hydrolase with wide substrate specificity from glycoside hydrolase family 97. J. Microbiol. Biotechnol..

[56] Jumper J., Evans R., Pritzel A., Green T., Figurnov M., Ronneberger O. (2021). Highly accurate protein structure prediction with AlphaFold. Nature.

[57] Koropatkin N.M., Martens E.C., Gordon J.I., Smith T.J. (2008). Starch catabolism by a prominent human gut symbiont is directed by the recognition of amylose helices. Structure.

[58] Cameron E.A., Maynard M.A., Smith C.J., Smith T.J., Koropatkin N.M., Martens E.C. (2012). Multidomain carbohydrate-binding proteins involved in *Bacteroides thetaiotaomicron* starch metabolism. J. Biol. Chem..

[59] Arnal G., Cockburn D.W., Brumer H., Koropatkin N.M. (2018). Structural basis for the flexible recognition of α-glucan substrates by *Bacteroides thetaiotaomicron* SusG. Protein Sci..

[60] Agirre J., Moroz O., Meier S., Brask J., Munch A., Hoff T. (2019). The structure of the AliC GH13 α-amylase from *Alicyclobacillus* sp. reveals the accommodation of starch branching points in the α-amylase family. Acta Crstallogr. D Struct. Biol..

[61] Andersen S., Møller M.S., Poulsen J.N., Pichler M.J., Svensson B., Lo Leggio L. (2020). An 1,4-α-glucosyltransferase defines a new maltodextrin catabolism scheme in Lactobacillus acidophilus. Appl. Environ. Microbiol..

[62] Ravcheev D.A., Godzik A., Osterman A.L., Rodionov D.A. (2013). Polysaccharides utilization in human gut bacterium *Bacteroides thetaiotaomicron*: comparative genomics reconstruction of metabolic and regulatory networks. BMC Genomics.

[63] Martens E.C., Lowe E.C., Chiang H., Pudlo N.A., Wu M., McNulty N.P. (2011). Recognition and degradation of plant cell wall polysaccharides by two human gut symbionts. PLoS Biol..

[64] Tamura K., Dejean G., Van Petegem F., Brumer H. (2021). Distinct protein architectures mediate species-specific beta-glucan binding and metabolism in the human gut microbiota. J. Biol. Chem..

[65] Koropatkin N., Martens E.C., Gordon J.I., Smith T.J. (2009). Structure of a SusD homologue, BT1043, involved in mucin O-glycan utilization in a prominent human gut symbiont. Biochemistry.

[66] Luis A.S., Briggs J., Zhang X., Farnell B., Ndeh D., Labourel A. (2018). Dietary pectic glycans are degraded by coordinated enzyme pathways in human colonic *Bacteroides*. Nat. Microbiol..

[67] Foley M.H., Martens E.C., Koropatkin N.M. (2018). SusE facilitates starch uptake independent of starch binding in *B. thetaiotaomicron*. Mol. Microbiol..

[68] Tauzin A.S., Kwiatkowski K.J., Orlovsky N.I., Smith C.J., Creagh A.L., Haynes C.A. (2016). Molecular dissection of xyloglucan recognition in a prominent human gut symbiont. mBio.

[69] Tamura K., Foley M.H., Gardill B.R., Dejean G., Schnizlein M., Bahr C.M.E. (2019). Surface glycan-binding proteins are essential for cereal beta-glucan utilization by the human gut symbiont *Bacteroides ovatus*. Cell Mol. Life Sci..

[70] Gray D.A., White J.B.R., Oluwole A.O., Rath P., Glenwright A.J., Mazur A. (2021). Insights into SusCD-mediated glycan import by a prominent gut symbiont. Nat. Commun..

[71] Dueñas-Chasco M.T., Rodríguez-Carvajal M.A., Tejero-Mateo P., Espartero J.L., Irastorza-Iribas A., Gil-Serrano A.M. (1998). Structural analysis of the exopolysaccharides produced by *Lactobacillus* spp. G-77. Carbohyr. Res..

[72] Dertli E., Colquhoun I.J., Gunning A.P., Bongaerts R.J., Le Gall G., Bonev B.B. (2013). Structure and biosynthesis of two exopolysaccharides produced by *Lactobacillus johnsonii* FI9785. J. Biol. Chem..

[73] Sarbini S.R., Kolida S., Naeye T., Einerhand A., Brison Y., Remaud-Simeon M. (2011). *In vitro* fermentation of linear and α-1,2-branched dextrans by the human fecal microbiota. Appl. Environ. Microbiol..

[74] Djouzi Z., Andrieux C., Pelenc V., Somarriba S., Popot F., Paul F. (1995). Degradation and fermentation of α-gluco-oligosaccharides by bacterial strains from human colon: *in vitro* and *in vivo* studies in gnotobiotic rats. J. Appl. Bacteriol..

[75] Miyamoto J., Shimizu H., Hisa K., Matsuzaki C., Inuki S., Ando Y. (2023). Host metabolic benefits of prebiotic exopolysaccharides produced by *Leuconostoc mesenteroides*. Gut Micobes.

[76] Ye J., Coulouris G., Zaretskaya I., Cutcutache I., Rozen S., Madden T.L. (2012). Primer-BLAST: a tool to design target-specific primers for polymerase chain reaction. BMC Bioinform..

[77] Somogyi M. (1952). Notes on sugar determination. J. Biol. Chem..

[78] Nelson N. (1944). A photometric adaptation of the Somogyi method for the determination of glucose. J. Biol. Chem..

[79] Kitaoka M. (2023). Automatic calculation of the kinetic parameters of enzymatic reactions with their standard errors using Microsoft Excel. J. Appl. Glycosci..

[80] Chiku K., Tsukasaki R., Teshima Y., Yoshida M., Aramasa H., Nihira T. (2020). Alkoxycarbonyl elimination of 3-*O*-substituted glucose and fructose by heat treatment under neutral pH. Carbohydr. Res..

[81] Kabsch W. (2010). XDS. Acta Crystallogr. D Biol. Crystallogr..

[82] Potterton L., Agirre J., Ballard C., Cowtan K., Dodson E., Evans P.R. (2018). CCP4i2: the new graphical user interface to the CCP4 program suite. Acta Crystallogr. D Biol. Crystallogr..

[83] Vagin A., Teplyakov A. (2010). Molecular replacement with MOLREP. Acta Crystallogr. D Biol. Crystallogr..

[84] Emsley P., Lohkamp B., Scott W.G., Cowtan K. (2010). Features and development of coot. Acta Crystallogr. D Biol. Crystallogr..

[85] Murshudov G.N., Skubák P., Lebedev A.A., Pannu N.S., Steiner R.A., Nicholls R.A. (2011). REFMAC5 for the refinement of macromolecular crystal structures. Acta Crystallogr. D Biol. Crystallogr..

[86] Holm L. (2020). DALI and the persistence of protein shape. Protein Sci..

[87] Varadi M., Anyango S., Deshpande M., Nair S., Natassia C., Yordanova G. (2022). AlphaFold protein structure database: massively expanding the structural coverage of protein-sequence space with high-accuracy models. Nucl. Acids Res..

