# Supplementary material for: Bacteroidota polysaccharide utilization system for branched dextran exopolysaccharides from lactic acid bacteria
Source: J Biol Chem. 2023 Jun 2;299(7):104885. doi: 10.1016/j.jbc.2023.104885 (PMC10316084; doi:10.1016/j.jbc.2023.104885)
Supplement: Supplemental data [file mmc1.pdf]

Supporting information

Bacteroidota polysaccharide utilization system for branched dextran exopolysaccharides from lactic acid bacteria

Shuntaro Nakamura[1], Rikuya Kurata[2], Takashi Tonozuka[3], Kazumi Funane[4], Enoch Y. Park[1,2,5], and Takatsugu Miyazaki[1,2,5*]

[1] Department of Bioscience, Graduate School of Science and Technology, Shizuoka University, 836 Ohya Suruga-ku, Shizuoka 422-8529, Japan

[2] Department of Agriculture, Graduate School of Integrated Science and Technology, Shizuoka University, 836 Ohya, Suruga-ku, Shizuoka, 422-8529, Japan.

[3] Department of Applied Biological Science, Tokyo University of Agriculture and Technology, 3-5-8 Saiwai-cho, Fuchu, Tokyo, 183-8509, Japan.

[4] Faculty of Life and Environmental Sciences, University of Yamanashi, 4-4-37, Takeda-cho, Kofu, Yamanashi, 400-8510, Japan.

[5] Research Institute of Green Science and Technology, Shizuoka University, 836 Ohya, Suruga-ku, Shizuoka, 422-8529, Japan.

***Correspondence:** Takatsugu Miyazaki, Research Institute of Green Science and Technology, Shizuoka University, 836 Ohya, Suruga-ku, Shizuoka, 422-8529, Japan; Tel.: +81-54-238-4886; E-mail: miyazaki.takatsugu@shizuoka.ac.jp

List of materials included

Figure S1. Expression levels of FjDexUL genes in the presence of different carbon sources.
Figure S2. Expression and purification of FjDexUL proteins.
Figure S3. General property of FjGH66 and FjGH97A.
Figure S4. Overall structure of FjGH66 and ligand-binding modes.
Figure S5. Crystal structure of FjGH65A in complex with IG2.
Figure S6. AlphaFold2 models of FjDusD and FjDusE.
Figure S7. Structural comparison of ligand-binding clefts of SusD superfamily proteins.
Figure S8. Sequence alignment of SusD superfamily proteins.
Figure S9. Sequence alignment of SusE_F superfamily proteins.
Figure S10. Isothermal titration calorimetry thermograms for FjDusD.
Table S1. Sequence of oligonucleotides used in this study.

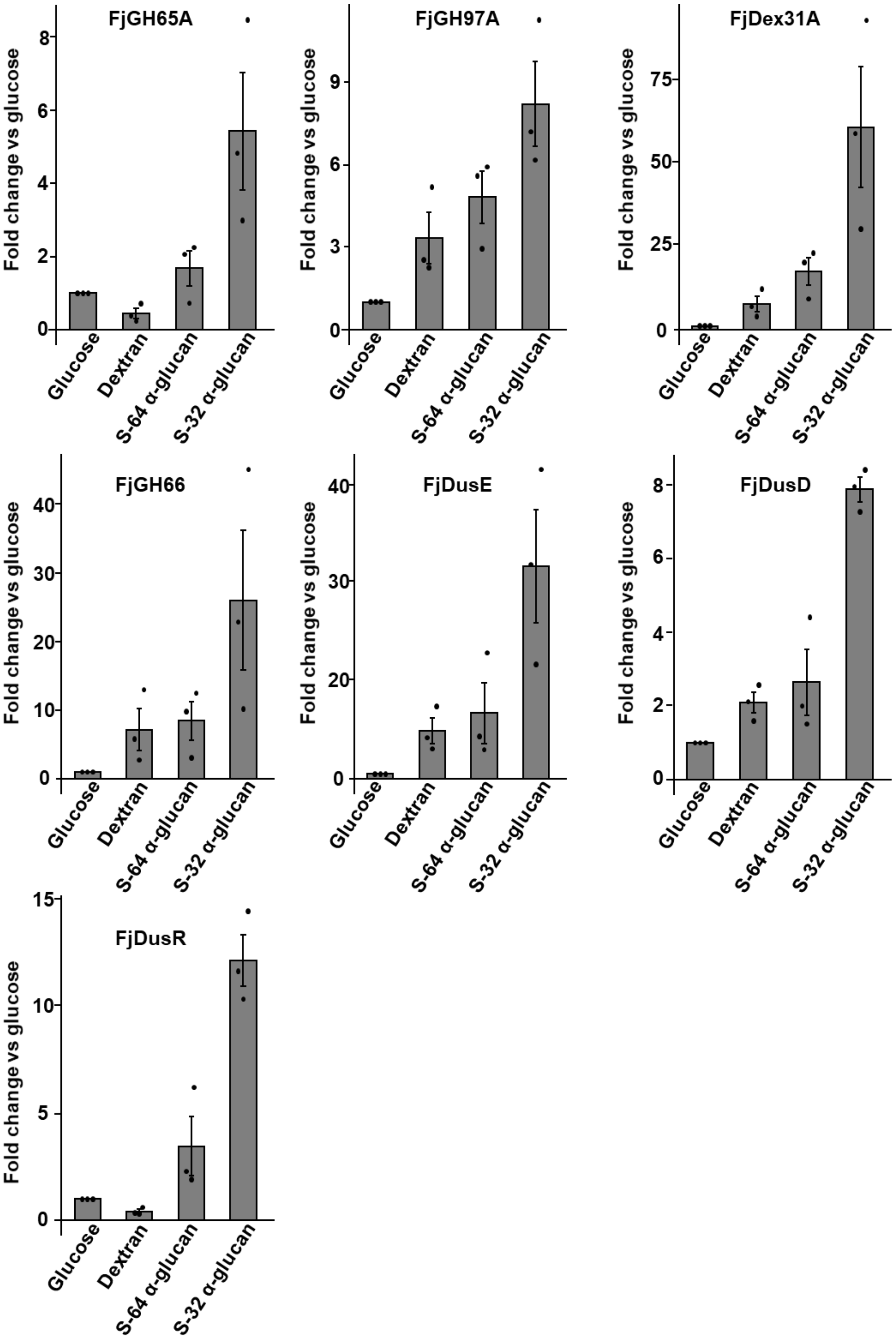

Figure S1. Expression levels of FjDexUL genes in the presence of different carbon sources. The expression levels of the FjDexUL genes in cells grown on glucans were normalized to that for glucose. The results are presented as means ± standard deviations of biological triplicates (n = 3).

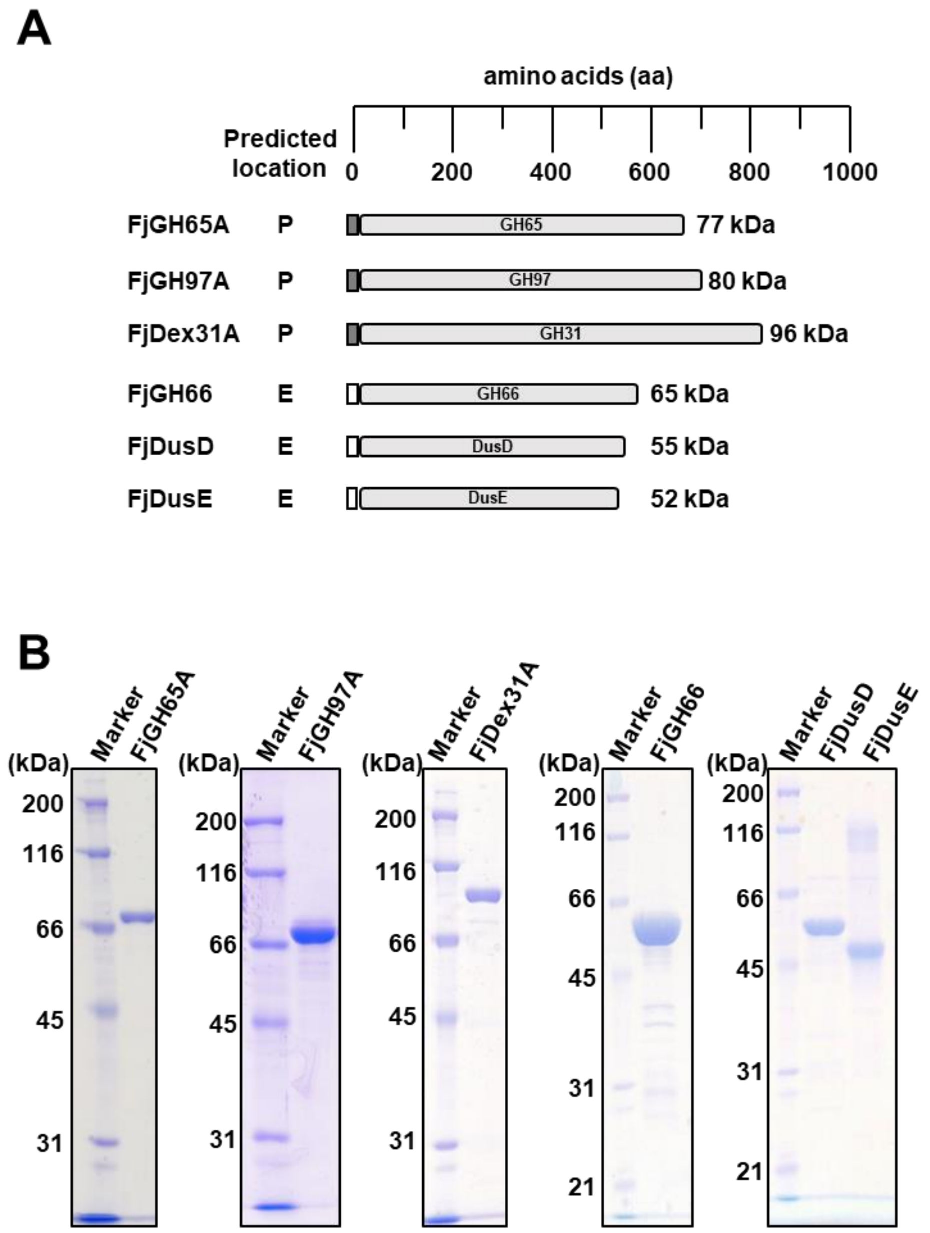

Figure S2. Expression and purification of FjDexUL proteins.

A, Molecular sizes of FjDexUL proteins. P and E indicate periplasmic and extracellular, respectively. B, SDS-PAGE analysis of purified FjDexUL proteins. A molecular weight marker is used for protein size determination (M. W. 6,500–200,000).

.

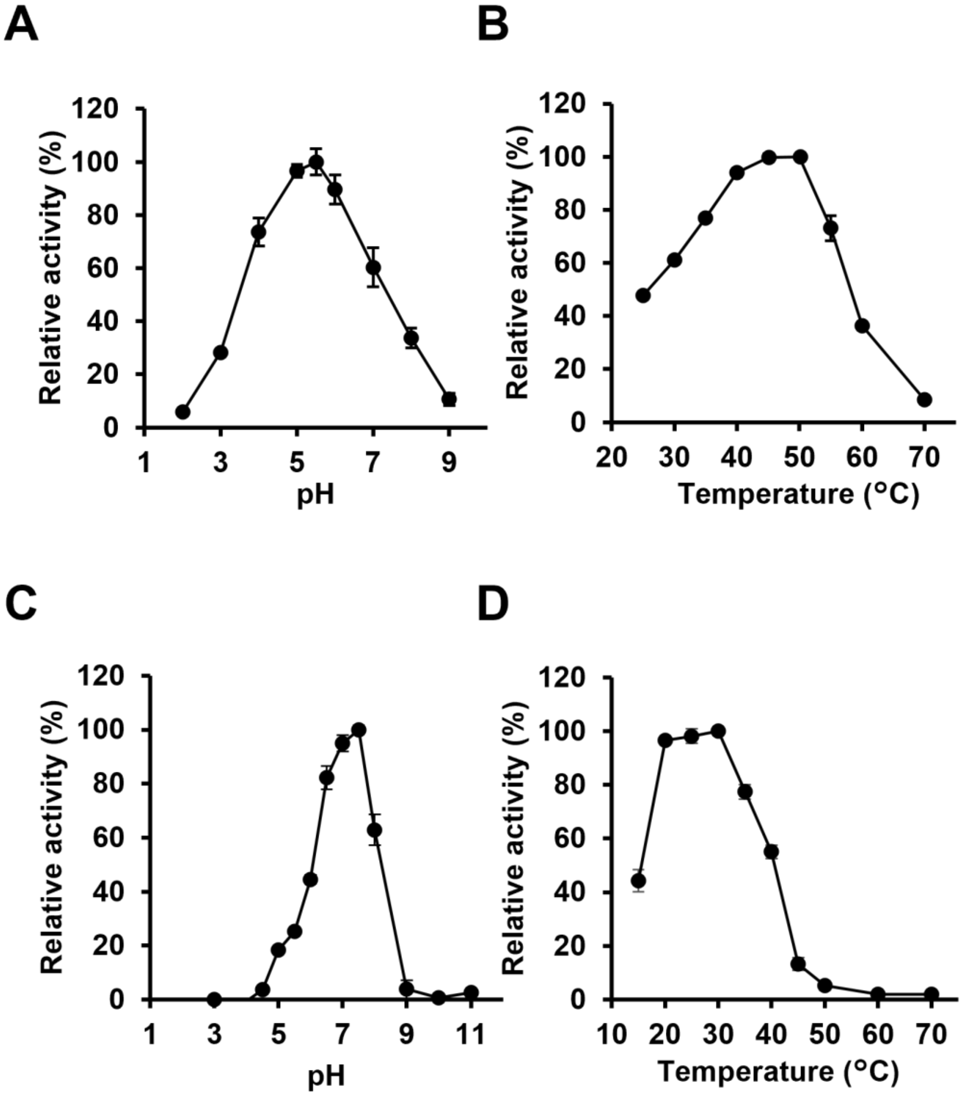

Figure S3. General property of FjGH66 and FjGH97A.

A, pH dependence for FjGH66-mediated hydrolysis of dextran 40,000 was measured at 30°C for 10 min using Britton–Robinson buffer (pH 2.0–9.0). C, Temperature dependence of FjGH66 hydrolysis for dextran 40,000 was measured at 25°C–70°C using 50 mM MES-NaOH buffer (pH 5.5). C, pH dependence of FjGH97A hydrolysis for pNP-Glc was measured at 30°C for 10 min using Britton–Robinson buffer (pH 2.0–9.0). D, Temperature dependence of FjGH97A hydrolysis for pNP-Glc was measured at 25°C–70°C using Britton–Robinson buffer (pH 7.0).

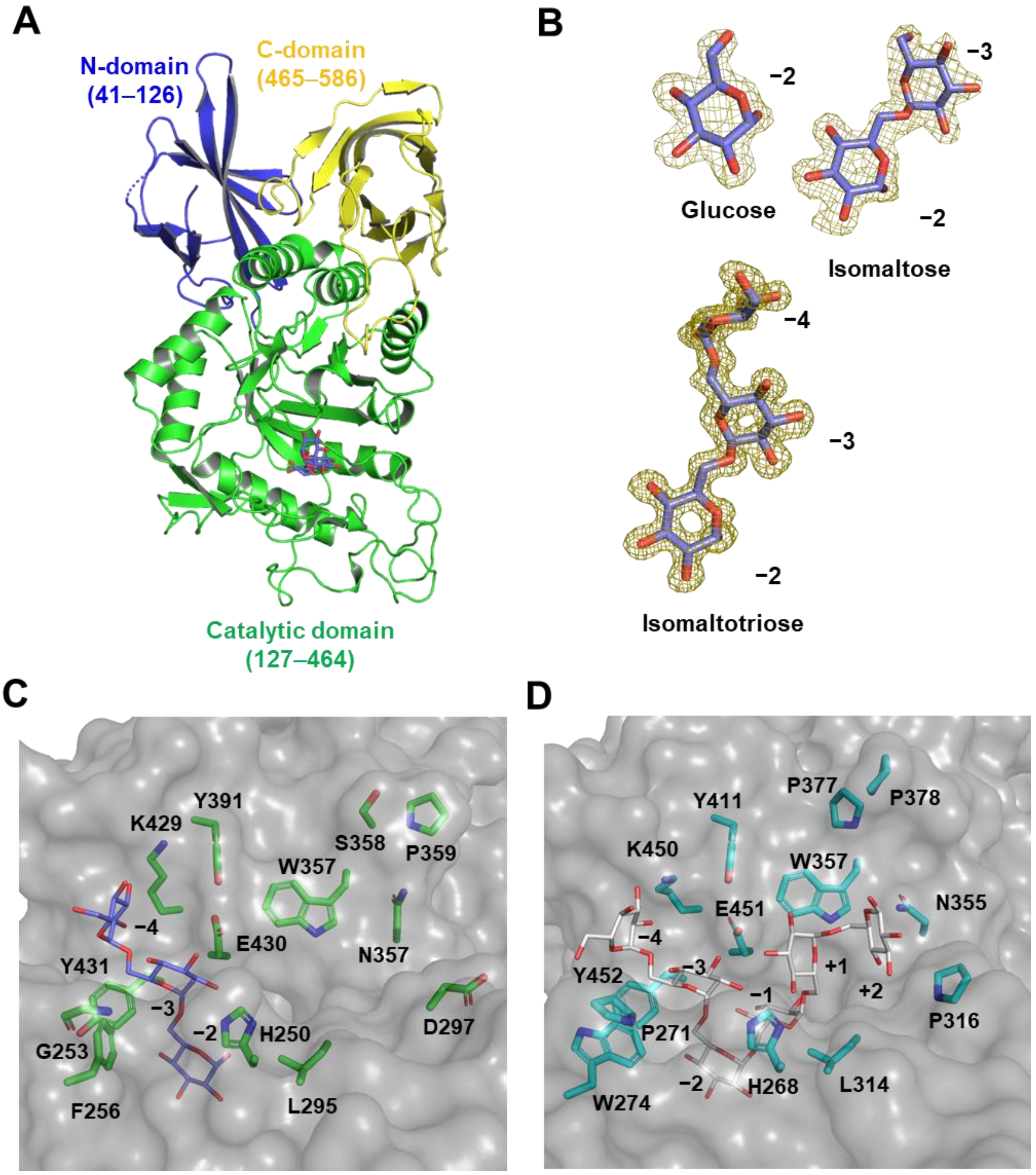

Figure S4. Overall structure of FjGH66 and ligand-binding modes.

A, Ribbon model of the FjGH66 monomer. The colors used are as follows: N-domain, blue; catalytic domain, *green*; and C-domain, *yellow*. The bound IG3 molecules are indicated as stick models in *slate blue*. B, $F_o - F_c$ omit electron density maps contoured at 3σ (*olive* mesh) for glucose, IG2, and IG3 (*slate blue* stick model) found in glucose-complex, IG2-complex, and IG3-complex, respectively. C and D, The surface model around the catalytic cleft of FjGH66 (C) and TpDex (D). FjGH66 and TpDex are colored in *gray*. *Slate blue* stick model, bund IG3; white stick model, bound IG6; the side chains of amino acid residues are shown in *green* (FjGH65A) and *cyan* (TpDex) stick models.

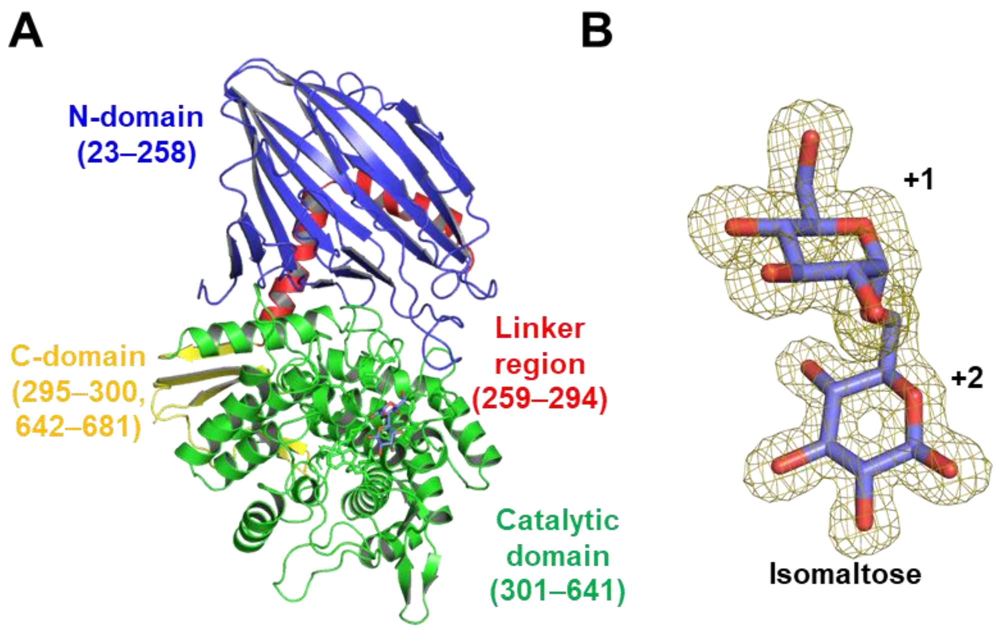

Figure S5. Crystal structure of FjGH65A in complex with IG2.

A, Ribbon model of the FjGH65A monomer. The colors used as follows: N-domain, *blue*; linker region, *red*; catalytic domain, *green*; and C-domain, *yellow*. B, $F_o - F_c$ omit electron density maps contoured at 3σ, and the IG2 model was shown as *olive* mesh and *slate blue* stick models, respectively.

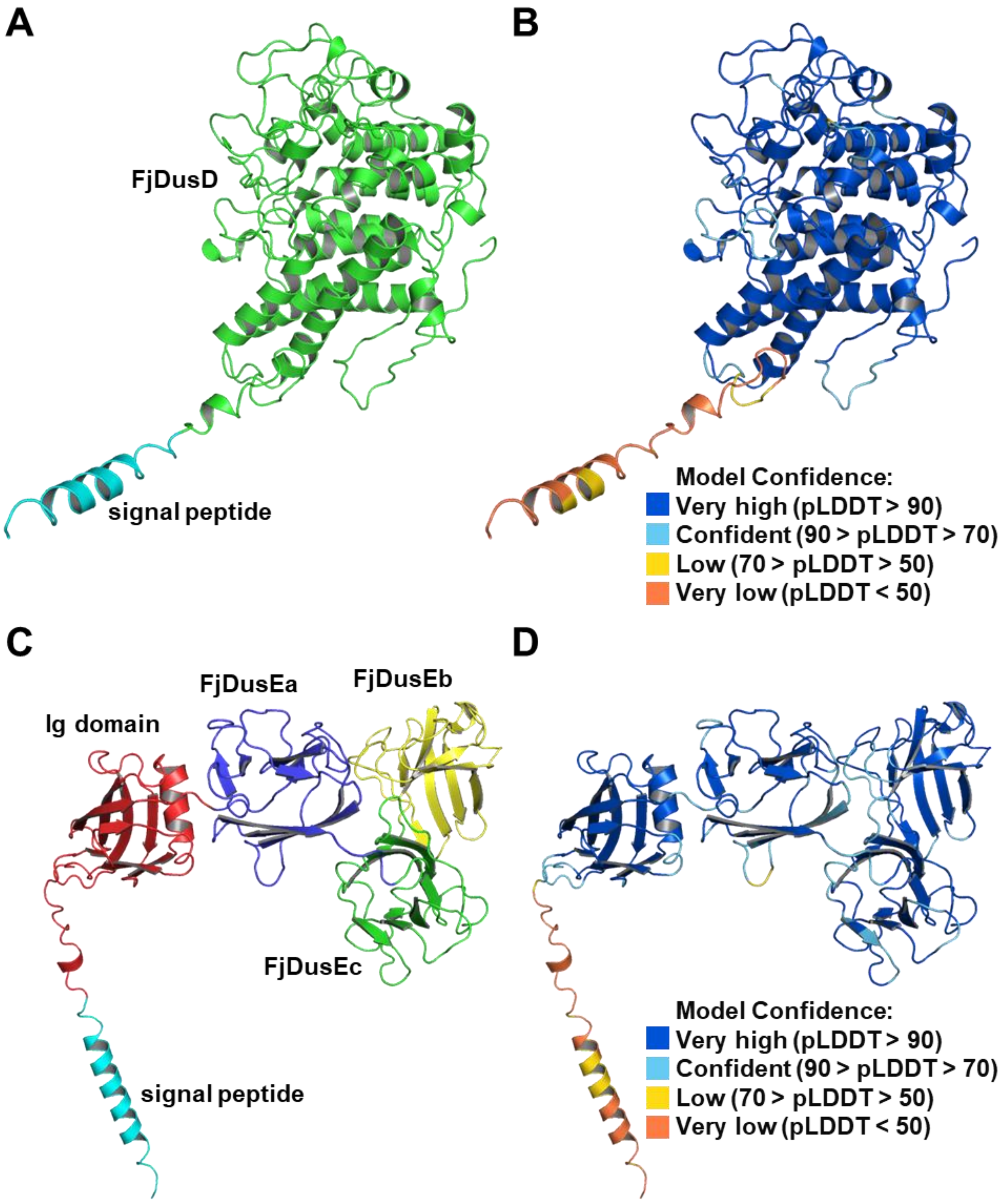

Figure S6. AlphaFold2 models of FjDusD and FjDusE.

A, Ribbon model of the FjDusD model (*green*). Signal peptide of FjDusD is shown in cyan. B, FjDusD model is colored according to the pLDDT score (<50 *orange*, <70 *yellow*, <90 *light blue*, >90 *dark blue*). C, Ribbon model of the FjDusE model. The colors used are as follows: Signal peptide, *cyan*; Ig-domain, *red*; FjDusEa, *blue*; FjDusEb, *yellow*; FjDusEc, *green*. D, FjDusE model is colored according to the pLDDT score (<50 *orange*, <70 *yellow*, <90 *light blue*, >90 *dark blue*).

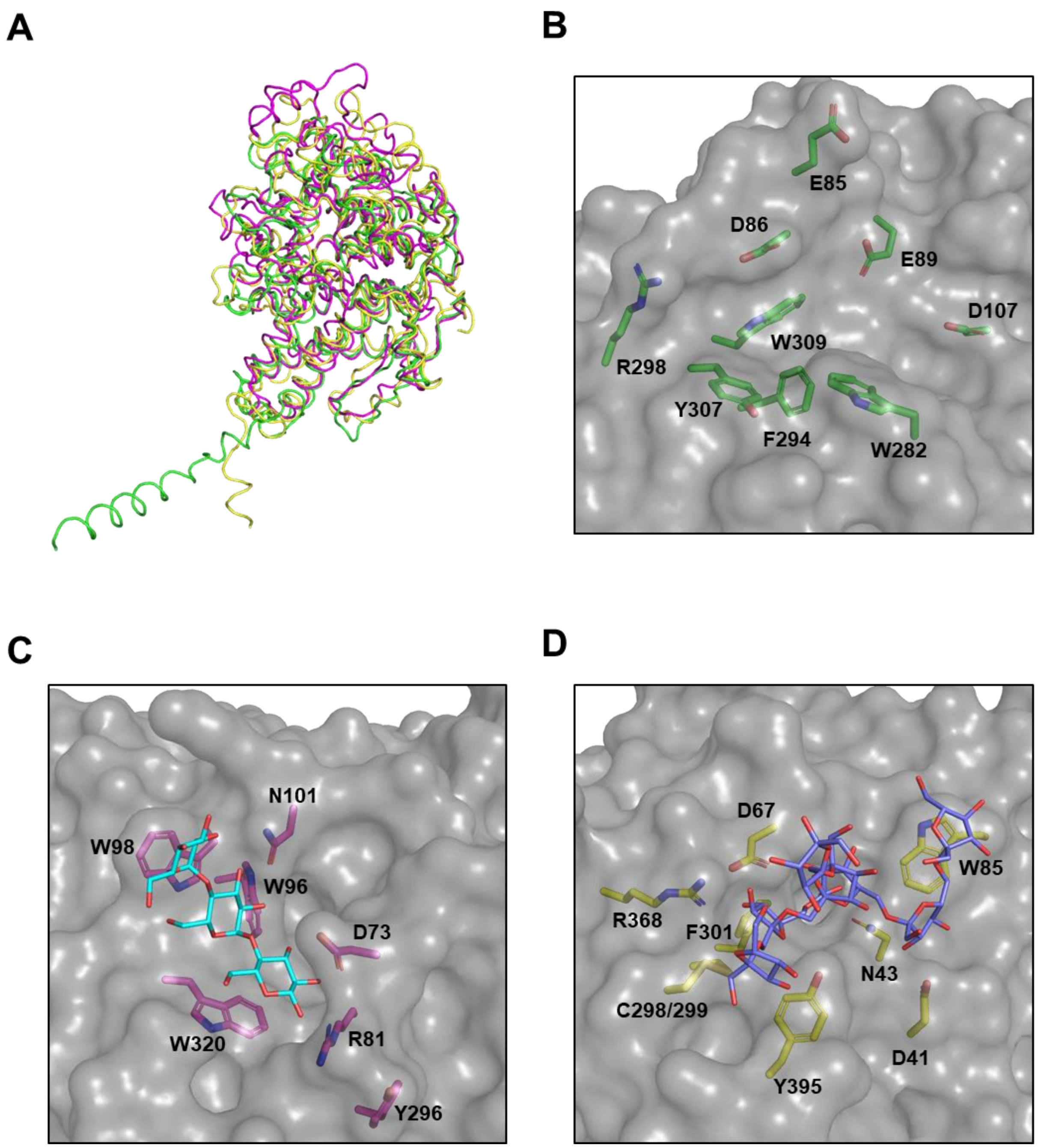

Figure S7. Structural comparison of ligand-binding clefts of SusD superfamily proteins.

A, Structural comparison with SusD superfamily proteins. Colors used are as follows: FjDusD Alphafold2 model, *green*; SusD (PDB 3CKC), *magenta*; BT1762 (PDB 6ZAZ), *yellow*. B–D, The ligand-binding clefts of FjDusD AlphaFold2 model (B), SusD (C), and BT1762 (D) are shown as molecular surface models (*gray*). *Cyan* stick model, bound maltotriose; *slate blue* stick model, bound β-D-fructofuranose-(2→6)-β-D-fructofuranose-(2→6)-β-D-fructofuranose-(2→6)-[β-D-fructofuranose-(2→1)]-β-D-fructofuranose-(2→6)-β-D-fructofuranose-(2→6)-β-D-fructofuranose; the side chains of amino acid residues are shown in *green* (FjDusD), *magenta* (SusD), and *yellow* (BT1762) stick models.

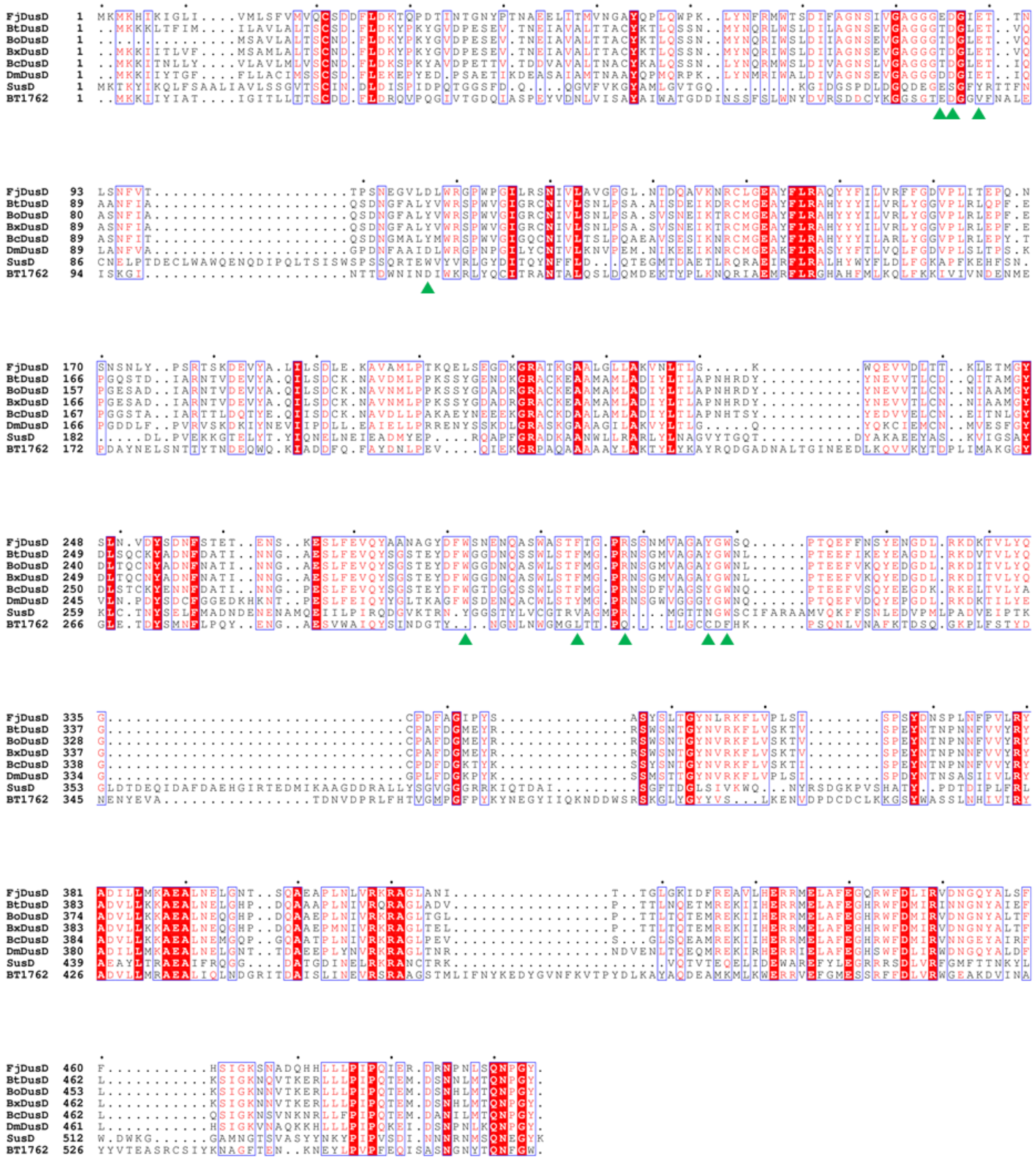

Figure S8. Sequence alignment of SusD superfamily proteins.

The sequence of SusD superfamily proteins, including FjDusD, closely-related homologs of Bacteroidota, SusD, and BT1762 were aligned using Clustal Omega. A figure was then generated using ESPript 3.0. Abbreviations: DusD, SusD homolog of dextran utilization locus; BtDusD, *Bacteroides thetaiotaomicron* DusD; BoDusD, *Bacteroides ovatus* DusD; BcDusD, *Bacteorides cellulosilycus* BFG-250 DusD; BxDusD, *Bacteroides xylanisolvens* XB1A DusD; DmDusD, *Dysgonomonas mossii* DSM 22836 DusD. Amino acid residues of FjDusD ligand-binding cleft are indicated as *green* triangles.

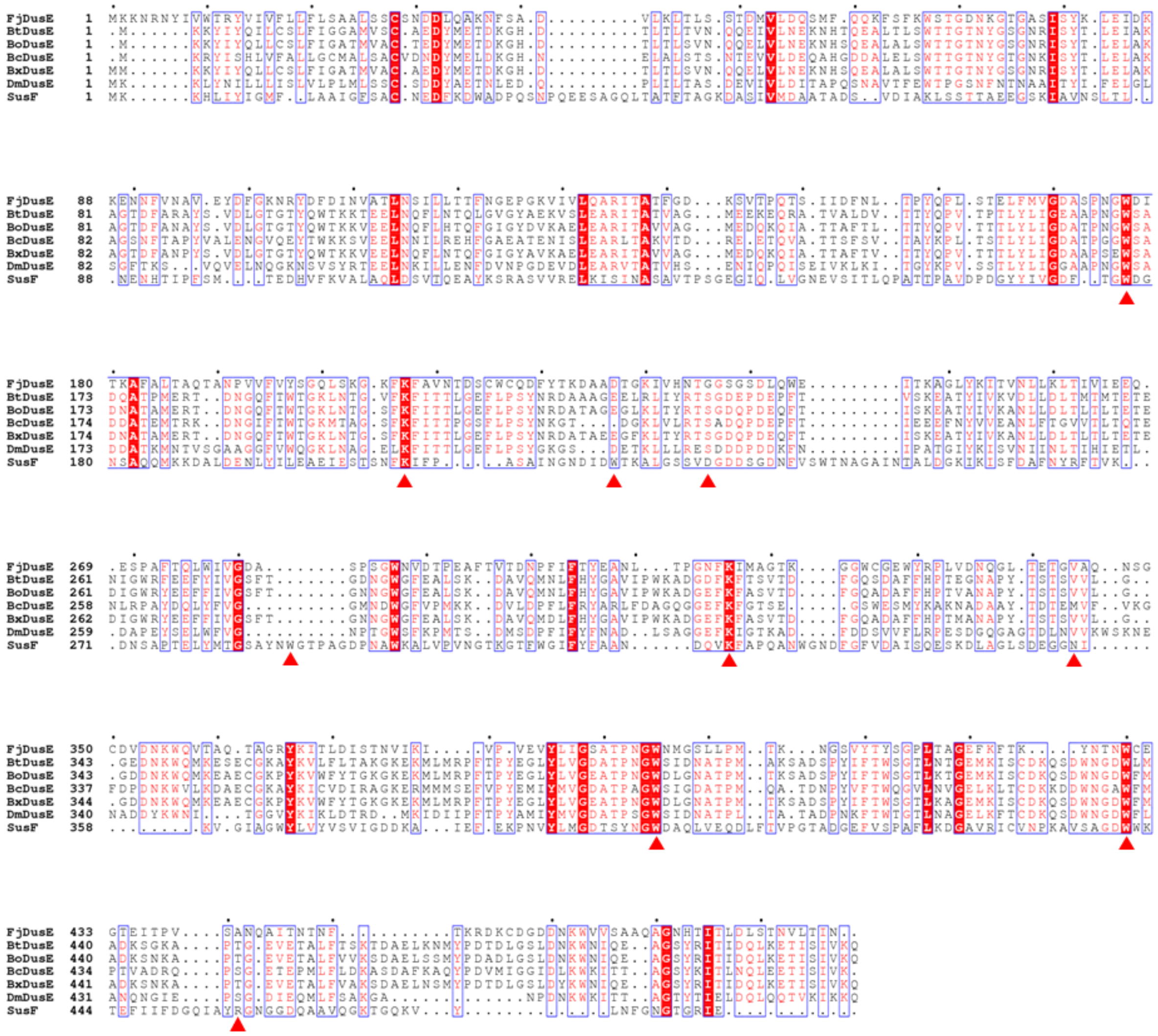

Figure S9. Sequence alignment of SusE_F superfamily proteins.

The sequence of SusE_F superfamily proteins, including FjDusE, closely-related homologs of Bacteroidota, and SusF were aligned using Clustal Omega. A figure was then generated using ESPript 3.0. Abbreviations: DusE, SusE/F homolog of dextran utilization locus; BtDusE, *Bacteroides thetaiotaomicron* DusE; BoDusE, *Bacteroides ovatus* DusE; BcDusE, *Bacteorides cellulosilycus* BFG-250 DusE; BxDusE, *Bacteroides xylanisolvens* XB1A DusE; DmDusE, *Dysgonomonas mossii* DSM 22836 DusE. Amino acid residues involved in ligand binding of SusF are indicated as *red* triangles.

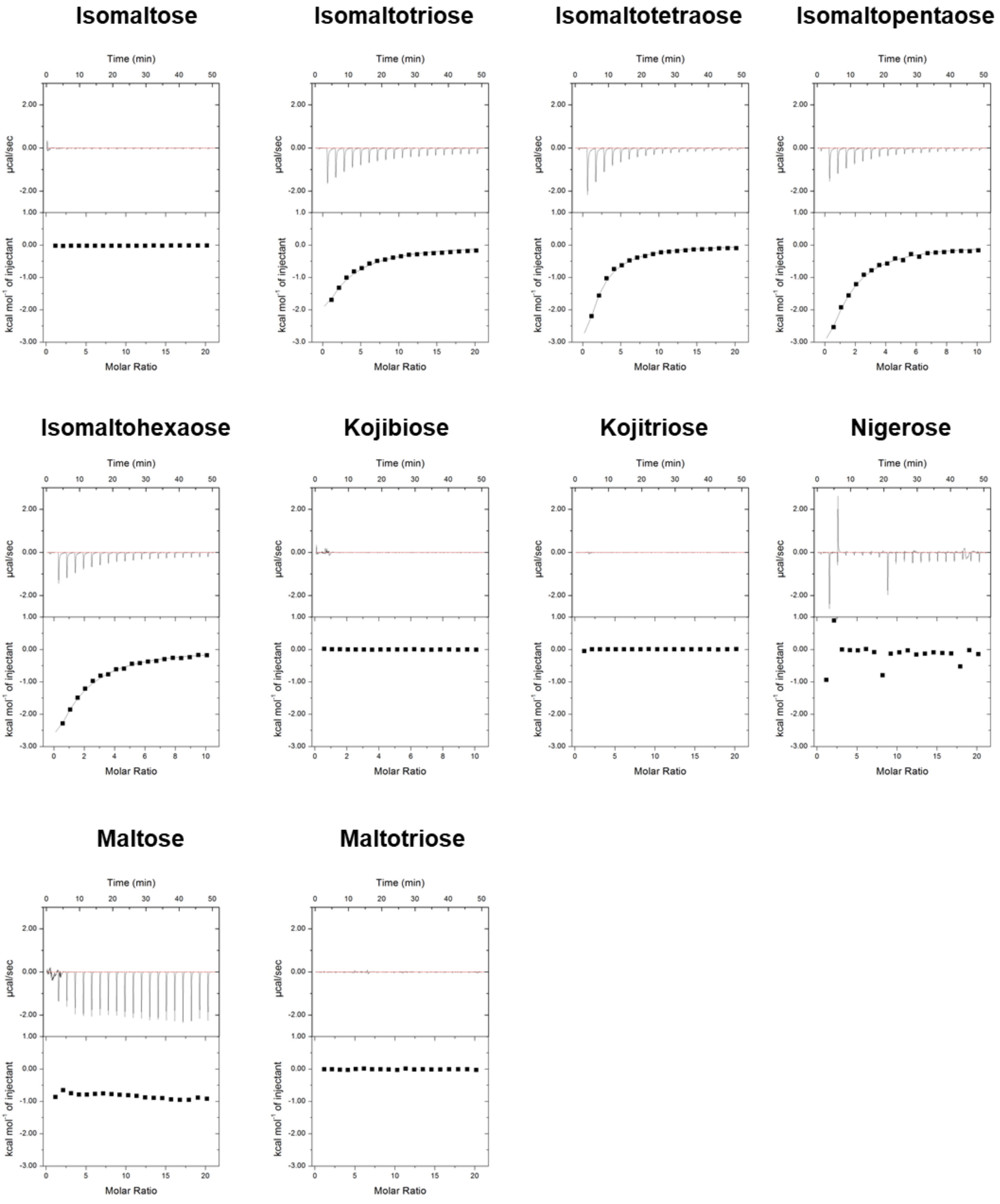

Figure S10. Isothermal titration calorimetry thermograms for FjDusD.

All titrations were performed in 10 mM HEPES-NaOH buffer (pH 7.0) at 25°C using 0.1 mM FjDusD. In each case, the upper graph shows the raw injection heat signal and the bottom graph shows the integrated heats. The detailed conditions are described in the Experimental Procedure section.

Table S1. Sequence of oligonucleotides used in this study.

Oligonucleotide	Sequence (5'→3') [a]
qRT-PCR	
Fj16SrRNA_F	GCAGGATGACGGTCCTATGG
Fj16SrRNA_R	CACGGAGTTAGCCGATCCTT
FjDusR_F	TGTTAGCCTCGAATAGGCGG
FjDusR_R	TGCAAGCGCTCATAGTGATCT
FjDusC_F	CGTCGGCTGTAATGTCCCAT
FjDusC_R	TGATGCTCATTTTGGCGCAC
FjDusD_F	TTGCGAACCAAATTGAGCGG
FjDusD_R	TTCCCGGTCCTTCGTTATGC
FjDusE_F	CTGTACCATTCTCCGCACCA
FjDusE_R	ATGTCGATACGCCAGAAGCC
FjGH66_F	GTCCTGCTTACGGATGCTGT
FjGH66_R	GAGGATTTCAGCTCCGCACT
FjDex31A_F	ACACCAAACTGCAGCCATCT
FjDex31A_R	GGGATGGGGCTAATTCCGTT
FjGH97A_F	GGAAACATGGCGCAACTACC
FjGH97A_R	TTCCCAGCCTTCGTTCCATC
FjGH65A_F	TGACCCTTCCACGCTTGTAG
FjGH65A_R	AGAAACGGTGGCTAATGGCA
Gene cloning	
FjGH66AΔ33-NheI-F	TTTTTGCTAGCGCCGAAGATGCAGTTACTG
FjGH66A-XhoI-R	TTTTTCTCGAGCTATTCAACTACAATCATT
FjGH97AΔ20-NheI-F	TTTTTGCTAGCGATTTAAAATCGCCTGACG
FjGH97A-XhoI-R	TTTTTCTCGAGTCACTTTTTTTTTGCTGCA
FjDusDΔ19-NheI-F	TTTTTGCTAGCAGTGATGATTTTTTAGATA
FjDusD-XhoI-R	TTTTTCTCGAGTTAGTAACCTGGATTTTGA
FjDusEΔ28-NheI-F	TTTTTGCTAGCAGCAATGATGATCTTCAGG
FjDusE-XhoI-R	TTTTTCTCGAGTTAGTTGATTGTAAGGACA

[a] Restriction sites are underlined.
